# FGA modulates immune infiltration and tumor progression via SLC7A11/xCT-mediated disulfidptosis in the tumor microenvironment of lung adenocarcinoma

**DOI:** 10.3389/fimmu.2025.1595900

**Published:** 2025-08-11

**Authors:** Gen Li, Qiuping Li, Sheng Yang, Dongmei Guo, Yanling Tao, Yan Jia

**Affiliations:** 1 Department of Wound Reconstructive Surgery, Tongji Hospital, School of Medicine, Tongji University, Shanghai, China; 2 Department of Clinical Medicine, Jining Medical University, Jining, China; 3 Department of Hematology, Affiliated Hospital of Jining Medical University, Jining, China; 4 Weishan County People’s Hospital, Jining, China

**Keywords:** immune infiltration, disulfidptosis, tumor microenvironment, non-small-cell lung cancer, lung adenocarcinoma

## Abstract

Emerging evidence highlights the tumor microenvironment’s (TME) crucial role in driving tumorigenesis and malignant progression. Disulfidptosis has recently been discovered as a non-apoptotic cell death mechanism triggered by intracellular disulfide stress. However, the impact of disulfidptosis within the dynamic modulation of the immune and stromal components in the TME of lung adenocarcinoma (LUAD) remains poorly characterized. In the presented study, RNA-seq and clinical data of LUAD patients were downloaded from TCGA; screening for genes associated with disulfidptosis and immune infiltration, revealed that fibrinogen alpha chain (FGA) modulates immune infiltration via disulfidptosis regulation. We also *in-vitro* experiments identified FGA suppression abrogates disulfidptosis through SLC7A11/xCT downregulation and attenuated disulfidptosis while concurrently enhancing malignant phenotypes, including cellular proliferation, migratory capacity, and invasive potential in LUAD models. This study reveals that FGA functions as a tumor suppressor that can impede the tumorigenesis of LUAD by modulating xCT expression, suggesting a novel therapeutic strategy enabling modulation of disulfidptosis for LUAD management.

## Introduction

Lung cancer has been the primary cause of cancer morbidity and mortality in recent years, with nearly one in eight (12.4%) cancers and one in five (18.7%) cancer deaths globally attributed to lung cancer (1). Lung cancer, the leading cause of cancer-related mortality, accounts for approximately 2.5 times the daily death toll of colorectal cancer, the second most common cause of cancer-related mortality (2). Lung adenocarcinoma (LUAD) is the most common subtype of lung cancer worldwide, according to a recent study (3). Stage I non-small-cell lung cancer (NSCLC) patients have an approximately 80% 5-year survival rate, while those with stages II to III disease have a 13%–60% 5-year survival rate. However, for stage IV patients, the 5-year survival rate is less than 10% (4). Surgical resection is the standard treatment for patients with stage I, stage II, and some stage IIIA disease. Despite advances in conventional therapies, current treatment modalities, including chemotherapy and radiation, continue to demonstrate limited efficacy in improving long-term survival outcomes for patients with advanced-stage NSCLC. Immunotherapy has not only proven effective in treating advanced carcinomas but has also become the standard treatment for NSCLC patients lacking actionable oncogenic mutations (5). In the last few years, the tumor microenvironment (TME) has become an area of intense research in NSCLC, particularly in LUAD, where further exploration remains imperative.

Normal cell types and cancer cells are interwoven in tumors through a complex network of blood vessels and extracellular matrix (6). Through intercellular communication, tumor cells create an environment that encourages cancer cells to survive and proliferate, and thus tumor growth. A recent study elucidated that molecular crosstalk in the tumor immune microenvironment may influence the nature of the tumor, but cancer cell mutations can modulate the recruitment and activation of immune cells, enabling cancer cells to develop mechanisms to resist immunotherapy through immune escape (7). Understanding the functions of cells within the tumor immune microenvironment is crucial for better comprehending the immune system’s role in tumor initiation and progression, as well as maximizing the potential of immunotherapy (8). In particular, the recent discovery of disulfidptosis, a novel cell death mechanism in SLC7A11-overexpressing cancer cells, provides new insights. This process is initiated by NADPH depletion, which leads to cystine accumulation, aberrant disulfide bond formation, and ultimately cytoskeletal collapse-induced cell death. One study, which analyzed the genetic profile of HCC patients, found that mutations in disulfidptosis-related genes occurred in 7.14% of patients. Moreover, patients with higher levels of disulfidptosis-related gene mutations exhibited increased immune infiltration and immunosuppression (9). Given these findings, disulfidptosis has emerged as a pivotal focus in cancer research.

Elevated reactive oxygen species (ROS) levels in cancer cells, driven by oncogenic signaling, compromised mitochondrial function, and hyperactive metabolism, paradoxically exert detrimental effects on malignant survival when exceeding cellular antioxidative capacity (10). To ensure cell survival and proliferation, cancer cells typically maintain sufficient glutathione (GSH) levels to neutralize ROS. Solute carrier family 7 member 11 (SLC7A11/xCT), which is overexpressed in most cancer cells, imports extracellular cystine and reduces it in the cytoplasm to cysteine with the help of glucose-derived nicotinamide adenine dinucleotide phosphate (NADPH), which serves as a precursor for the synthesis of GSH, thus enabling antioxidant defense (11, 12). However, glucose starvation induces NADPH depletion, which can cause an abnormal accumulation of cystine and other disulfide molecules in the cell, which ultimately results in a state of disulfide stress (13). Notably, myeloid cells had the greatest capacity to absorb intratumoral glucose, followed by T cells and tumor cells, across various cancer models (14). Recent evidence demonstrates that disulfidptosis plays a critical role in CD8+ T-cell exhaustion by inducing disulfide stress in tumor-infiltrating CD8+ T cells, ultimately leading to impaired antitumor immunity (15). Currently, TME and disulfidptosis offer a distinctive direction for cancer therapy, so understanding the role of disulfidptosis in tumor and immune infiltration is crucial. Our results reveal that the TME may regulate immune infiltration and disulfidptosis through the fibrinogen alpha chain (FGA).

Fibrinogen, fibrin, and their degradation products are involved in blood clotting, inflammation, and angiogenesis. However, recent studies have shown FGA, determined as both a candidate coding and non-coding driver, regulates hepatocellular carcinoma progression and metastasis (16). Here, our study showed a tumor suppressor role of FGA in LUAD. FGA suppresses LUAD progression by inducing disulfidptosis through xCT regulation and enhancing immune infiltration. This study identifies novel therapeutic strategies for lung cancer treatment.

## Methods

### Data sources and preprocessing

Transcriptome profiling data with clinical information were obtained from the TCGA-LUAD project by R (version 4.4.1) with the R package TCGAbiolinks. The inclusion of 513 primary solid tumor cases included intact clinical information (age, sex, T stage, N stage, M stage, and prognostic information) and 58 normal samples.

Read counts per gene were generated using HTSeq-count and used as input for pairwise differential expression analysis with DESeq2; the threshold values were |log_2_FoldChange| > 1 and adjusted *P*-value < 0.05. TPMs were transformed to log_2_(TPM+1) for further analyses. The R package maftools was used to perform the mutation spectrum analysis. Patient simple nucleotide variation data (MuTect2) retrieved through the R package maftools. Waterfall plots were created using the ComplexHeatmap package. Mutational burden is calculated by multiplying the number of mutations by the number of bases covered in a sample and is reported as mutations per megabase.

### Immune infiltration analysis

Estimate is a method used in tumor samples to determine the proportions of stromal and immune cells by analyzing gene expression patterns. We applied it to assess the TME of each LUAD patient, along with stromal score (stromal content), immune score (extent of immune cell infiltration), ESTIMATE score (synthetic mark of stroma and immune), and tumor purity using the R package estimate (17).

CIBERSORT was applied to calculate immune cell composition from gene expression profiles. This deconvolution algorithm was used to estimate the abundance of different immune cell populations from expression data (18).

The level of infiltration of immune cell types in the R package gsva was analyzed by using ssGSEA. The expression levels of genes in 28 published gene sets for immune cells were used to calculate the extent of infiltration of 28 immune cell types (19).

### Survival analysis

Survival analysis was performed using R (version 4.4.1) with the packages survival and survminer. Survival was plotted using a Kaplan–Meier survival curve, and statistical significance was determined by the log-rank (Mantel-Cox) test. *p* < 0.05 was considered significant.

### Consensus clustering based on FLNA and NDUFS1

Expressions of FLNA and NDUFS1 were extracted and clustered coherently using the R package ConsensusClusterPlus. The samples were divided into two clusters. Heatmaps were produced by the R language with the package pheatmap.

### Gene set enrichment analysis

The C5 gene set was used to download gene sets for canonical pathways and Gene Ontology from the Molecular Signatures Database (MSigDB). GSEA was conducted with the R package clusterProfiler to identify the significant functional difference between the two clusters. Significant pathway enrichment was identified by the normalized enrichment score (|log_2_FoldChange| >1), *p*-value < 0.05, and FDR *q*-value < 0.05.

### Weighted gene co-expression network analysis

Weighted Gene Co-Expression Network Analysis (WGCNA) was performed using the R package WGCNA. To ensure that the constructed co-expression networks are more in line with the characteristics of scale-free networks, we chose 7 as the soft power. We got 12 modules and determined the correlations between them and cluster, stromal score, immune score, ESTIMATE score, and tumor purity.

### GO enrichment analysis

GO enrichment analyses were performed with R packages clusterProfiler, enrichplot, and ggplot2. Only terms with both *p*- and *q*-values of <0.05 were considered significantly enriched.

### PPI network construction

The PPI network was constructed using the STRING database with a minimum interaction score of medium confidence (0.400), followed by reconstruction using Cytoscape (version 3.10.2).

### Cell lines, reagents, and antibodies

Human LUAD epithelial cells (A549 and NCI-H1299) were obtained from the Cell Bank of the Chinese Academy of Sciences (Shanghai, China) and cultured in DMEM (low glucose) (Gibco, Shanghai, China), supplemented with 10% fetal bovine serum (FBS) (Gibco). A humidified atmosphere with 5% CO_2_ was used to maintain human cells at 37°C and test for mycoplasma contamination. Cell lines were regularly examined to confirm morphological and growth characteristics that indicate that cell line. In this study, the following antibodies and reagents were employed: β-actin (Abcom), FGA (Sigma-Aldrich, Abcom), xCT (Proteintech), goat anti-rabbit secondary antibody (Abcom), Ki-67 (Abcom), and Alexa 488–labeled secondary antibody (Abcam).

### Plasmids, siRNA, and transfection

Overexpression plasmids were constructed by Genechem. The following siRNA targeting FGA was used: siFGA: 5′-UUUGUAUUUGUGAAGAUGCtt-3′; Lipofectamine 3000 (Invitrogen) was used to transfect cells with the indicated plasmids and siRNAs in accordance with the manufacturer’s instructions.

### RNA isolation and real-time q-PCR

Total RNA was extracted by using the SPARKeasy Cell RNA Kit (Sparkjade). Reverse transcription was performed using a cDNA synthesis kit (Thermo Fisher Scientific). Primer sequences were as follows: FGA, forward:5′- GTCTTCTTTGCTAGAGAAGTGGAGA-3′, reverse: 5′- AAAGAATGTTTCTCTTGCCTTCCTG-3′. GAPDH, forward:5′-GAAGGTGAAGGTCGGAGTC-3′, reverse: 5′-GAAGATGGTGATGGGATTTC-3′. Relative expression levels were determined by normalizing the expression level of each target to GAPDH, and relative mRNA fold changes were determined using the 2^−ΔΔCt^ method.

### Immunofluorescence staining

Immunofluorescence staining for Ki-67 to evaluate cell proliferation, following the manufacturer’s instructions. Cells were incubated with a primary Ki-67 antibody (Abcam, USA; 1:300), followed by a secondary Alexa 488–labeled antibody (Abcam, USA; 1:1000) for 1h at room temperature. Subsequently, cells were counterstained with 10 mg/ml DAPI.

### CCK-8 assay and migration, invasion, and wound-healing assay

A cell proliferation assay was performed using the Cell Counting Kit-8 (CCK-8; beyotime). Transfected with siRNA or plasmid, cells were seeded in 96-well plates and incubated at 37°C for 24h; each well was incubated with 100 μl DMEM (low glucose) (Gibco) supplemented with 10 μl CCK-8 reagent at 37°C for 2h. The absorbance of every well was measured at 450 nm using a microplate reader (Thermo Fisher Scientific, Waltham, MA, USA).

For cell invasion and migration assays, cells were serum-starved for 24h pre-plating, and 2.5 × 104 cells were seeded in with or without Matrigel-coated transwell inserts (8-μm pore size, Corning). Briefly, cells in 100 μl serum-free media were added into the upper chamber, and 500 μl culture media with 10% FBS was in the lower well. After incubation for 24h, cells that remained on the upper surface of the membrane were removed with a cotton swab, and those on the underside of the membrane were fixed with 4% paraformaldehyde and stained with 0.5% crystal violet and microscopically counted from three random fields of each membrane. Carefully wounding confluent cell layers in six-well plates with a sterile 200 µl tip, washing them twice with medium, and culturing them for 48h. At a specific time, the area between the wound edges was measured before and after the recovery and quantified using light microscopy and ImageJ (version 1.54).

### Measurement of ROS

Intracellular ROS levels were measured using the CM-H2DCFDA assay kit (Beyotime Biotechnology, Shanghai, China) as per the manufacturer’s instructions. Treated or control adherent cells (incubated in low glucose medium) were washed with PBS. Cells were then incubated *in situ* with 5 µM CM-H2DCFDA diluted in the assay diluent provided with the kit for 30 min at 37°C in the dark. After washing the cells with PBS, they were analyzed using a flow cytometer (Agilent NovoCyte). The resulting data were processed using FlowJo software.

### Xenotransplantation assays

All animal experiments were conducted in accordance with accepted standards of animal care and approved by the Institutional Animal Care and Use Committee of Affiliated Hospital of Jining Medical University (Jining, China). Seven-week-old female BALB/c nude mice were obtained from Shandong Pengyue (Shandong, China) and randomly divided into two groups. The mice were subcutaneously injected into the right flank with logarithmic-phase A549 cells (8 × 10^6^ cells per mouse) transfected with shFGA or Ctrl-GFP to establish a subcutaneous lung cancer xenograft model. Tumor size was measured every other day using calipers, and volume (V) was calculated as V = (L × W²)/2, where L is the longest diameter and W is the perpendicular shorter diameter.

### Co-immunoprecipitation

Co-IP assays were performed using Classic Magnetic Protein A/G IP Kits (Epizyme, Shanghai, China). Briefly, protein lysates were incubated with anti-FGA or anti-xCT (mouse monoclonal) antibodies for 1h, followed by incubation with pre-washed magnetic beads for an additional hour. After magnetic separation for 1 min, the bead-antibody-protein complexes were washed four times with lysis buffer to remove nonspecific interactions. Finally, the immunoprecipitated proteins were separated by SDS-PAGE for downstream analysis.

### Co-culture of transfected A549 cells with immune cells

A non-contact co-culture system was established using 0.4-μm pore Transwell inserts (Corning). A549 cells and CD8^+^ T cells were co-cultured at a 1:1 ratio. Transfected A549 cells (2 × 10^5^ cells/well) were seeded in six-well plates. Pre-activated CD8^+^ T cells (2 × 10^5^ cells/insert) were added to the Transwell inserts. Cells were maintained in RPMI-1640 medium supplemented with 10% FBS and 100 IU/ml IL-2. After 48h, CD8^+^ T cells from the inserts were collected for PCR analysis.

### Statistical analysis

Statistical analysis was performed using GraphPad Prism 9.0.0 (GraphPad Software, Inc.) and is shown as mean ± SD as indicated. Replicates are indicated in figure legends. The equality of variance was determined through an *F*-test. In samples that were normally distributed, a two-tailed t-test was used to compare the means of the variables between two groups. In samples with nonnormal distributions, the medians of the variable between two groups were compared by a Mann–Whitney *U* test. For analysis of more than two groups, we used the analysis of variance (ANOVA) to determine the equality of variance. Comparisons between groups were performed with Tukey–Krammer *post-hoc* analysis. For all tests, *P* < 0.05 was considered statistically significant.

## Results

### Scores exhibited a significant correlation with LUAD patient survival

**Figure 1:**
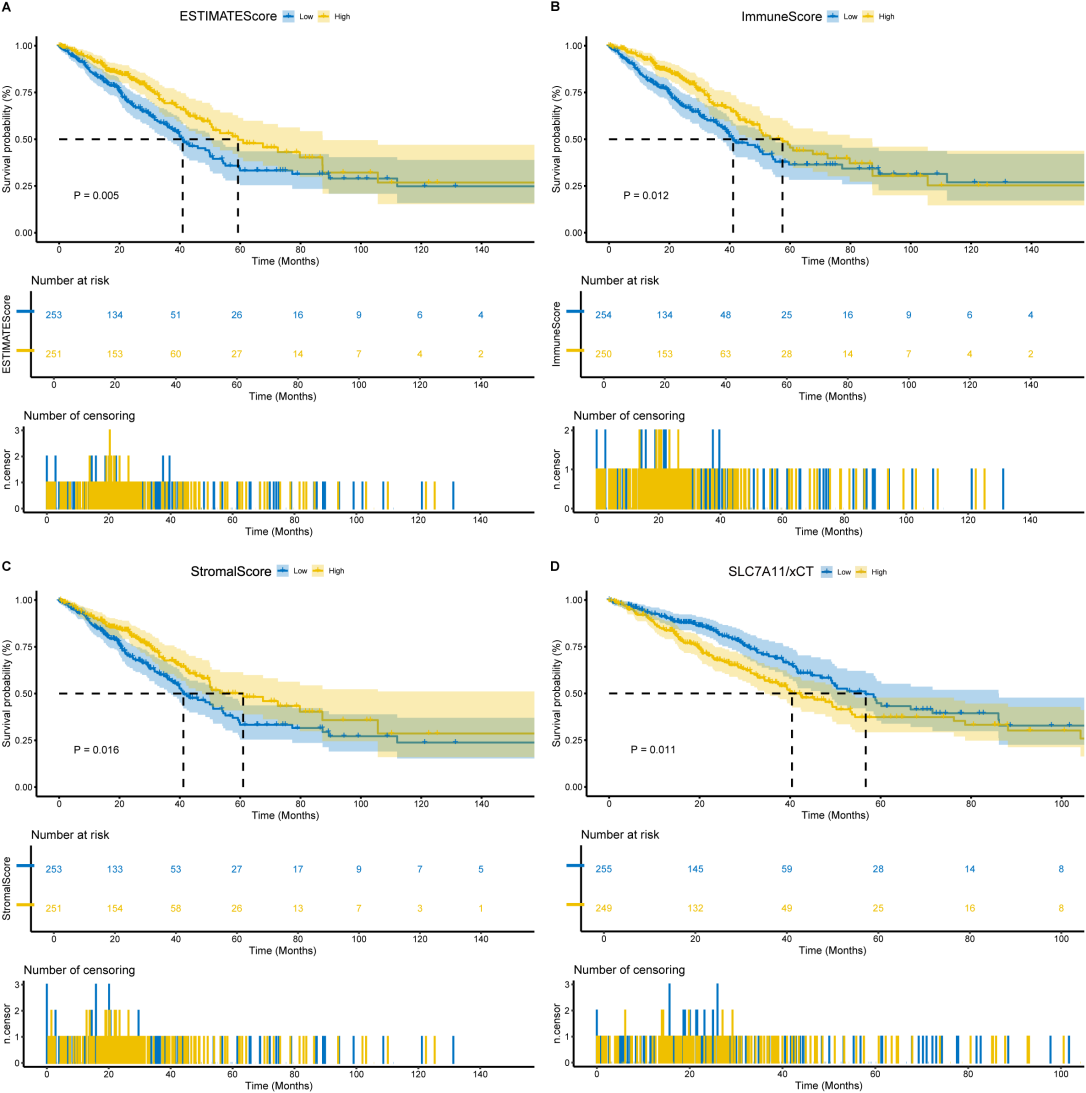
Association of scores with the survival of LUAD patients. **(A)** Kaplan–Meier survival analysis for LUAD patients grouped into high or low score in ESTIMATEScore determined by the comparison with the median. *p* = 0.005 by log-rank test. **(B)** Kaplan–Meier survival curve for ImmuneScore with *p* = 0.012 by log-rank test. **(C)** Kaplan–Meier survival curve for StromalScore with *p* = 0.016 by log-rank test. **(D)** Kaplan–Meier survival analysis for LUAD patients grouped into xCT high or low expression determined by the comparison with the median. *p* = 0.011 by log-rank test.

To determine the relationship between the estimated proportions of immune and stromal cells with the survival rate, we performed Kaplan–Meier survival analysis with 95% confidence for ImmuneScore, StromalScore, and ESTIMATEScore, respectively. The ESTIMATE algorithm by the R package ESTIMATE (20, 21) was used to assess the immune and stromal components in the TME for each sample. This assessment yielded three scores: ImmuneScore, StromalScore, and ESTIMATEScore. These scores were positively correlated with the proportions of immune, stromal, and the sum of both, respectively. Therefore, higher scores indicated a larger proportion of the corresponding components in the TME. As illustrated in **Figures 1A–C**, elevated ImmuneScore, StromalScore, and ESTIMATEScore demonstrated significant associations with improved overall survival rate (*p* < 0.05). According to these findings, the immune and stromal components in TME were effective predictors of prognosis in patients with LUAD.

### Scores were correlated with the clinicopathological staging of LUAD patients

**Figure 2:**
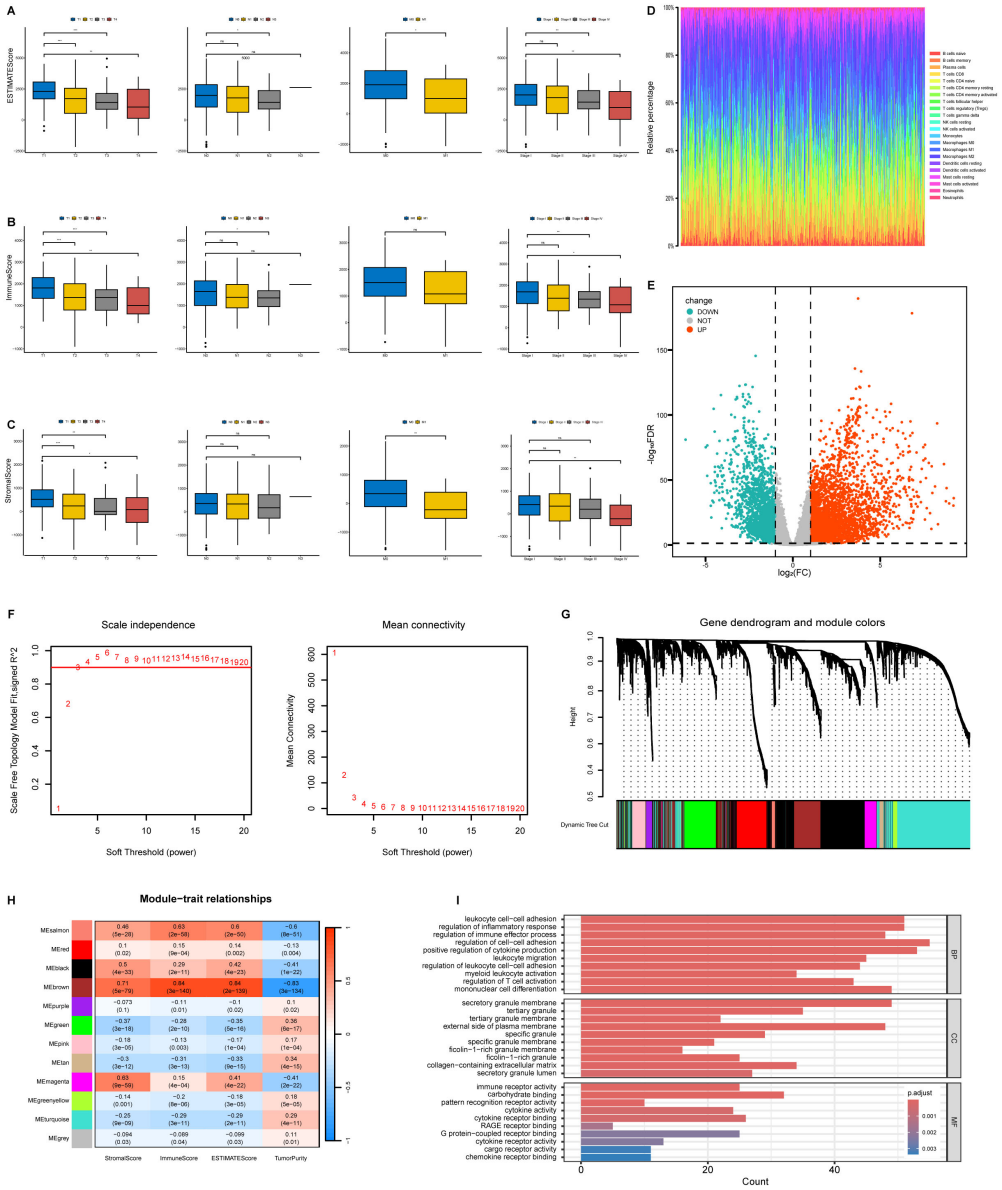
Correlation of ESTIMATE results with clinicopathological staging characteristics and identification of module genes associated with both clustering and immunity in the WGCNA. **(A)** Distribution of ESTIMATEScore in T classification, N classification, M classification and stage. **(B)** Distribution of ImmuneScore in T classification, N classification, M classification and stage. **(C)** Distribution of StromalScore in T classification, N classification, M classification and stage. **(D)** Barplot showing the proportion of 22 types of TICs in LUAD tumor samples. Column names of plot were sampled ID. **(E)** Volcano plot of differential analysis. **(F)** Analysis of network topology for soft powers. **(G)** Gene dendrogram and module colors. **(H)** Heatmap between module Eigen genes and ESTIMATE results. **(I)** The GO analysis of hub genes. ns, no significance, *P < 0.05, **P < 0.01, ***P < 0.001.

We analyzed the clinicopathological data from TCGA-LUAD cases to investigate associations between the proportions of immune and stromal components with their clinicopathological characteristics. ESTIMATE score demonstrated progressive decline with advancing TNM stage (**Figure 2A**). Immune Score showed inverse correlations with T classification, N classification, and overall stage (**Figure 2B**). StromalScore exhibited negative associations with T, M classification, and overall stage, but not N classification (**Figure 2C**). These findings indicate that altered immune-stromal compositional ratios correlate with advanced LUAD progression, particularly in mediating invasive and metastatic processes.

### WGCNA and identification of hub genes related to immunity

To characterize the immune landscape, we applied the CIBERSORT algorithm to quantify tumor-infiltrating subsets, generating 22 kinds of immune cell profiles in LUAD samples (**Figure 2D**). Comparative transcriptomic analysis of LUAD versus normal tissues in TCGA identified 5,404 differentially expressed genes (DEGs, |log_2_FC|>1, FDR < 0.05), and results were visualized using a volcano plot (**Figure 2E**). The genes were then considered for the WGCNA (**Figures 2F, G**). To identify a module related to immunity, we performed a correlation between modules and traits (**Figure 2H**). Notably, the brown module tended to be significantly associated with immunity (*R* = 0.84, *P* = 3e-140) (**Figure 2H**). We conducted Gene Ontology (GO) enrichment analysis on the brown module genes, revealing significant enrichment (FDR < 0.05) in biological processes including regulation of cell–cell adhesion and positive regulation of cytokine production, cellular components such as secretory granule membrane and the external side of the plasma membrane, as well as molecular functions encompassing immune receptor activity and carbohydrate binding (**Figure 2I**).

### Identification of immune-related disulfidptosis-related genes and consensus clustering of patients

**Figure 3:**
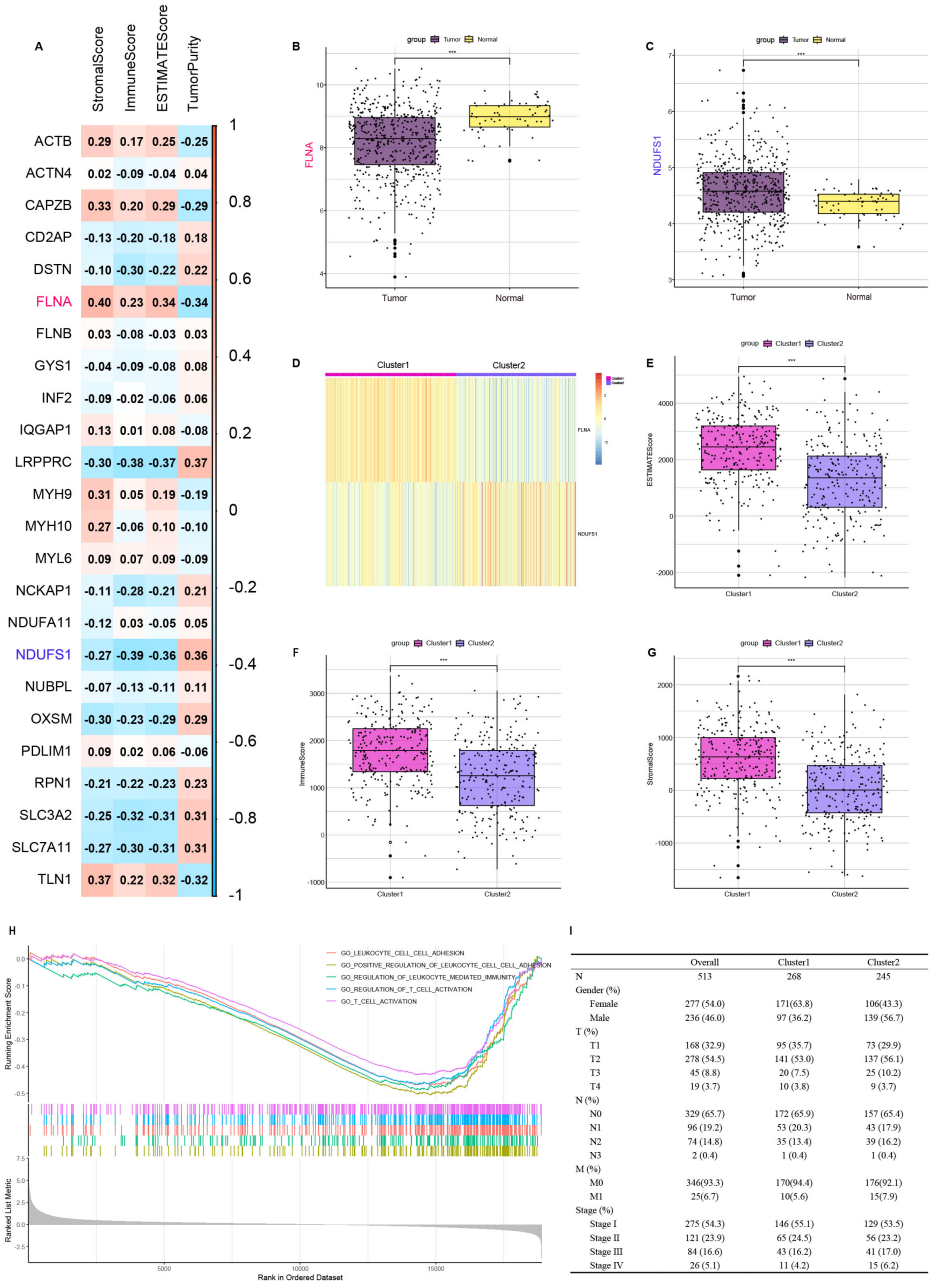
Identify disulfidptosis-related genes that influence ImmuneScore and cluster TCGA-LUAD patients based on the expression of FLNA and NDUFS1. **(A)** Association between disulfidptosis-related genes and results of ESTIMATE. **(B)** Comparison of FLNA expression between tumor and normal tissues. **(C)** Comparison of NDUFS1 expression between tumor and normal tissues. **(D)** TCGA-LUAD patients are divided into two clusters according to FLNA and NDUFS1. **(E)** Comparison of ESTIMATE score between two clusters. **(F)** Comparison of ImmuneScore between two clusters. **(G)** Comparison of StromalScore between two clusters. **(H)** Comparison of functional enrichment between two clusters. **(I)** Clinical features of two clusters. ***P < 0.001.

To delineate the immunomodulatory role of disulfidptosis in LUAD, 24 disulfidptosis-related genes were identified, and correlations between these genes and ESTIMATE scores were evaluated (**Figure 3A**). FLNA and NDUFS1 were included in the next analysis based on the highest absolute value of the correlation with the immune score. RNA-seq data from the TCGA-LUAD cohort were analyzed using the Wilcoxon rank-sum test, demonstrating significantly lower FLNA expression levels (*p* < 0.001) and elevated NDUFS1 expression (*p* < 0.001) in tumor tissues compared to normal counterparts (**Figures 3B, C**). Consensus clustering of 513 TCGA-LUAD samples based on FLNA and NDUFS1 expression stratified two clusters. The heatmap shows Cluster 1 (*n* = 268) has high expression of FLNA and low expression of NDUFS1, while Cluster 2 (*n* = 245) had low expression of FLNA and high expression of NDUFS1 (**Figure 3D**).

### Immune microenvironment profiling and functional annotations

ESTIMATE and GSEA were performed to understand the differences in immunological function better. In ESTIMATE analysis, Cluster 1 demonstrated higher stromal, immune, and ESTIMATE scores along with lower tumor purity than Cluster 2 (**Figures 3E–G**). To functionally annotate inter-cluster expression differences, we conducted Gene Set Enrichment Analysis (GSEA) using the c5.all.v7.0.Symbols.gmt (version 7.0 of the Molecular Signatures Database) reference set from the Molecular Signatures Database (v7.0). Our analysis incorporated all differentially expressed genes between Cluster 2 and Cluster 1. Multiple immune-related pathways showed significant enrichment, including leukocyte cell–cell adhesion, positive regulation of leukocyte cell–cell adhesion, regulation of leukocyte-mediated immunity, regulation of T-cell activation, and T-cell activation (**Figure 3H**). Clinical and tumor pathologic features of patients are summarized in **Figure 3I**.

### Comparison of immune infiltration and evaluation of sensitivity to immunotherapy and genetic mutation

**Figure 4:**
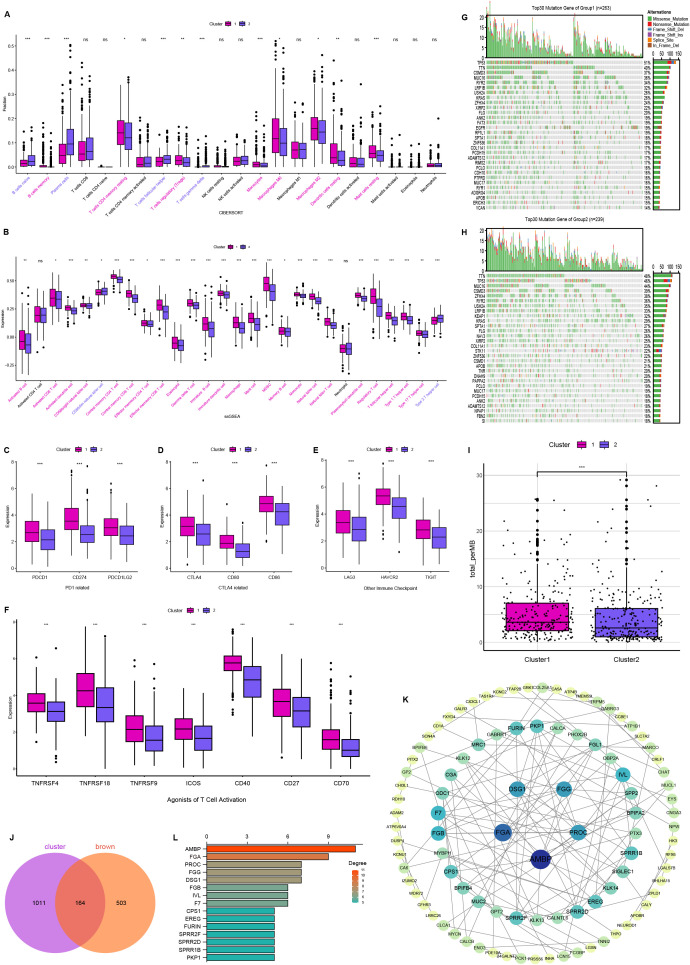
Comparison of immune and mutational landscapes’ characteristics between two clusters, and analysis of hub genes. **(A)** The proportion of immune cells between two clusters. **(B)** The expression of immune cells between two clusters. **(C–F)** Targets of immunomodulatory drugs between two clusters. Mutational landscape of Cluster 1 **(G)** and Cluster 2 **(H)**. **(I)** Comparison of tumor mutation burden (TMB) between two clusters. **(J)** Venn plot showing the common 164 genes shared by differentially expressed genes and brown module. **(K)** Interaction network constructed with the nodes with interaction confidence value > 0.95. **(L)** The top 15 genes ordered by the number of nodes. ns, no significance, *P < 0.05, **P < 0.01, ***P < 0.001.

We conducted CIBERSORT and ssGSEA analyses to characterize immune infiltration heterogeneity between the two clusters. CIBERSORT analysis demonstrated that Cluster 1 exhibited significantly higher proportions of B-cell memory, T-cell CD4 memory resting, T-cell regulatory (Tregs), monocytes, macrophages M0, macrophages M2, dendritic cells resting, and mast cells resting compared to Cluster 2 (**Figure 4A**). The ssGSEA further identified 28 immune cell subtypes, including activated B cells, activated CD8 T cells, activated dendritic cells, mast cells, macrophages, natural killer cells, and natural killer T cells, highly expressed in cluster 1 (**Figure 4B**). Next, we compared the expression levels of immunomodulatory targets in the two clusters. Most targets, including PD1, PDL1, PDL2, CTLA4, CD80, CD86, LAG3, TIM3, TIGHT, OX40, GITR, 4-1BB, ICOS, CD27, and CD70, were significantly higher in Cluster 1 (**Figures 4C–F**). Landscapes of mutation profiles between the two clusters are depicted (**Figures 4G, H**). Cluster 1 had higher TMB than Cluster 2 (**Figures 4I**). These results suggest that Cluster 1 had a stronger immune infiltration and a stronger response to immunotherapy than Cluster 2.

### Interaction analysis of differentially expressed genes

To identify a disulfidptosis and immunity-associated module, we analyzed the intersection between WGCNA-derived brown module genes and inter-cluster differentially expressed genes, yielding 164 consensus genes (**Figure 4J**). The protein-protein interaction (PPI) network was constructed using STRING (Search Tool for the Retrieval of Interacting Genes/Proteins) and visualized through Cytoscape v3.10.2, with nodes representing proteins and edges indicating functional associations (**Figure 4K**). Based on network topology analysis, genes were prioritized by node connectivity, with the 15 most interconnected candidates selected for further investigation (**Figure 4L**). These genes were subsequently advanced for functional validation studies.

### FGA knockdown promotes malignant phenotype and *in-vivo* growth of human lung adenocarcinoma cells

**Figure 5:**
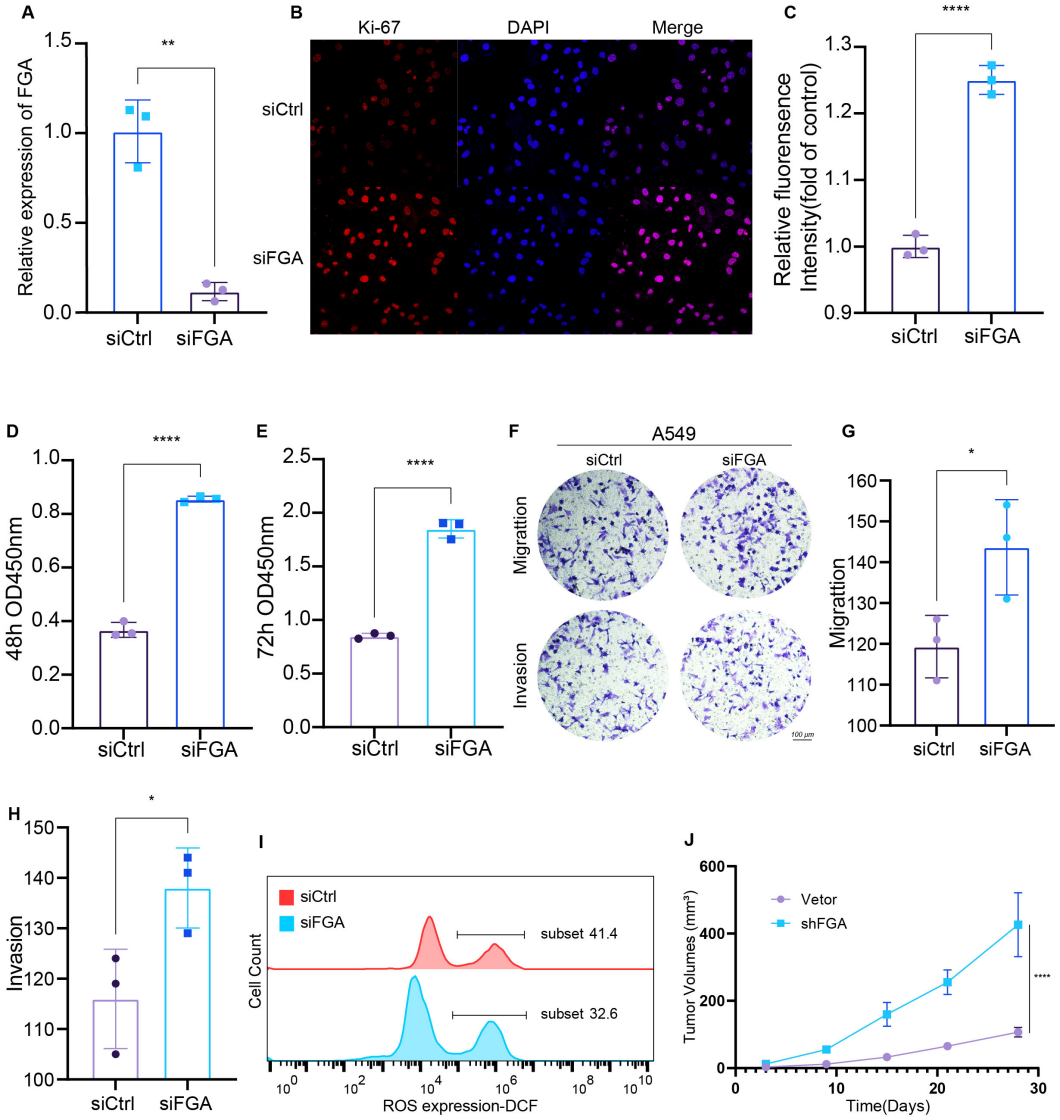
Functional Validation of FGA and Identification of Its Regulatory Role in Tumor Phenotypes. **(A)** Knockdown was confirmed by RT‐qPCR. Ki-67 staining **(B)** and quantification **(C)**. Proliferation of A549 cells transfected with siCtrl or siFGA after 48h **(D)** and 72h **(E)** was detected by CCK8. **(F–H)** Transwell migration and invasion assays using A549 cells transfected with siFGA or control siRNA. **(I)** Flow Cytometric Analysis of Intracellular Reactive Oxygen Species (ROS). **(J)** Tumor growth curves in nude mice. *P < 0.05, **P < 0.01, ****P < 0.0001.

Based on our literature mining and analysis of relevant studies, we identified FGA as a potential target gene and have decided to focus our research efforts on exploring the functional significance role of FGA in LUAD. A549 cells were cultured in DMEM (low glucose) and transfected with either control small interfering RNA (siCtrl) or FGA-targeting small interfering RNA (siFGA). To verify the effectiveness of these knockdowns, we analyzed RNA prepared from cells 24h after transfection by qPCR. mRNA expression of RNAi targets was efficiently reduced (**Figure 5A**).

Cell proliferation was first assessed through Ki-67 immunofluorescence labeling, revealing significantly elevated proliferative activity in siFGA-treated cells versus controls (**Figures 5B, C**). Subsequently, we performed an assessment of the effect of transfection on cell proliferation at 48 (**Figure 5D**) and 72h (**Figure 5E**) using a CCK8 assay. The results indicated that the knockdown of FGA significantly stimulated the proliferation of A549 cells in comparison to the siCtrl cells. Transwell migration and invasion assays demonstrated that siFGA significantly increased the number of migrating and invading cells (**Figures 5F–H**). To investigate whether siFGA reduces intracellular reactive oxygen species (ROS) levels, thereby decreasing glutathione (GSH) consumption and inhibiting disulfide bond formation and subsequent disulfidptosis, we measured intracellular ROS levels 24h post-transfection using the ROS Assay Kit (CM-H2DCFDA probe and Diluent). The results demonstrated that siFGA significantly reduced ROS levels (**Figure 5I**).

To assess the impact of FGA on tumor growth *in vivo*, tumor growth kinetics were evaluated in 7-week-old female immunodeficient BALB/c nude mice. Mice were subcutaneously inoculated with shFGA-expressing A549 cells or vector control A549 cells. Results showed that compared to tumors derived from vector control cells, xenograft tumors from shFGA A549 cells exhibited significantly accelerated growth (**Figure 5J**). These findings demonstrate that FGA suppresses LUAD progression *in vivo*. Collectively, these results suggest that FGA potentially functions as a tumor suppressor in LUAD via disulfidptosis.

### FGA functions as a tumor suppressor in LUAD through xCT

**Figure 6:**
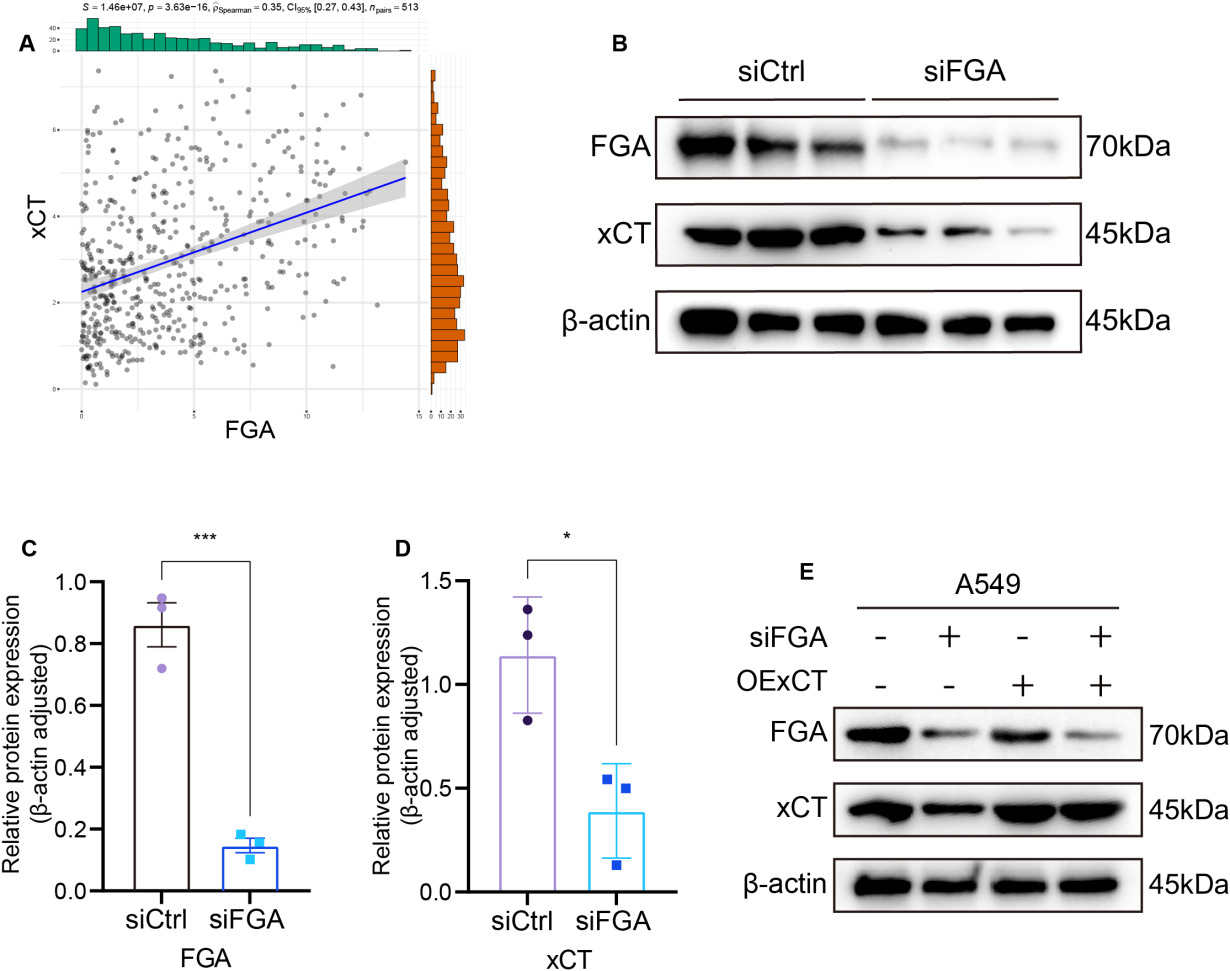
Identification of FGA regulatory targets. **(A)** Association between FGA and xCT expression. **(B)** Western blot assay of FGA and xCT expression in A549 cells transfected with siCtrl or siFGA. **(C)** FGA Relative protein expression (β-actin adjusted). **(D)** xCT Relative protein expression (β-actin adjusted). **(E)** Functional validation through rescue assays with concurrent Western blot analysis in A549 cells. *P < 0.05, ***P < 0.001.

The association between FGA and disulfidptosis was explored through Spearman’s rank correlation analysis across disulfidptosis-related gene sets. This revealed a statistically significant positive correlation between FGA and xCT (*R* = 0.35, *P* = 3.63e−16) (**Figure 6A**). Survival analysis revealed a negative correlation between xCT expression and overall survival (*P* = 0.011) (**Figure 1D**). Western blotting demonstrated that FGA-knockdown cells showed reduced xCT protein expression compared to siCtrl-transfected cells at 48h post-transfection (**Figure 6B**). Densitometric quantification of protein bands using ImageJ software confirmed significant downregulation of both FGA and xCT in the experimental group (**Figures 6C, D**).

To delineate whether FGA exerts tumor suppressive effects via xCT-mediated pathways, we conducted rescue experiments by transfection of xCT overexpression plasmid (OExCT). LUAD cells were co-transfected with either siCtrl or siFGA, along with either a control vector or OExCT. There were four experimental groups: the siCtrl and vector co-transfection group, the siFGA and vector co-transfection group, the siCtrl and OExCT co-transfection group, and the siFGA and OExCT co-transfection group. We found that transfection of the xCT overexpression plasmid significantly restored xCT protein expression in siFGA-transfected cells (**Figure 6E**).

### FGA suppresses lung cancer progression through modulation of xCT-mediated disulfidptosis

**Figure 7:**
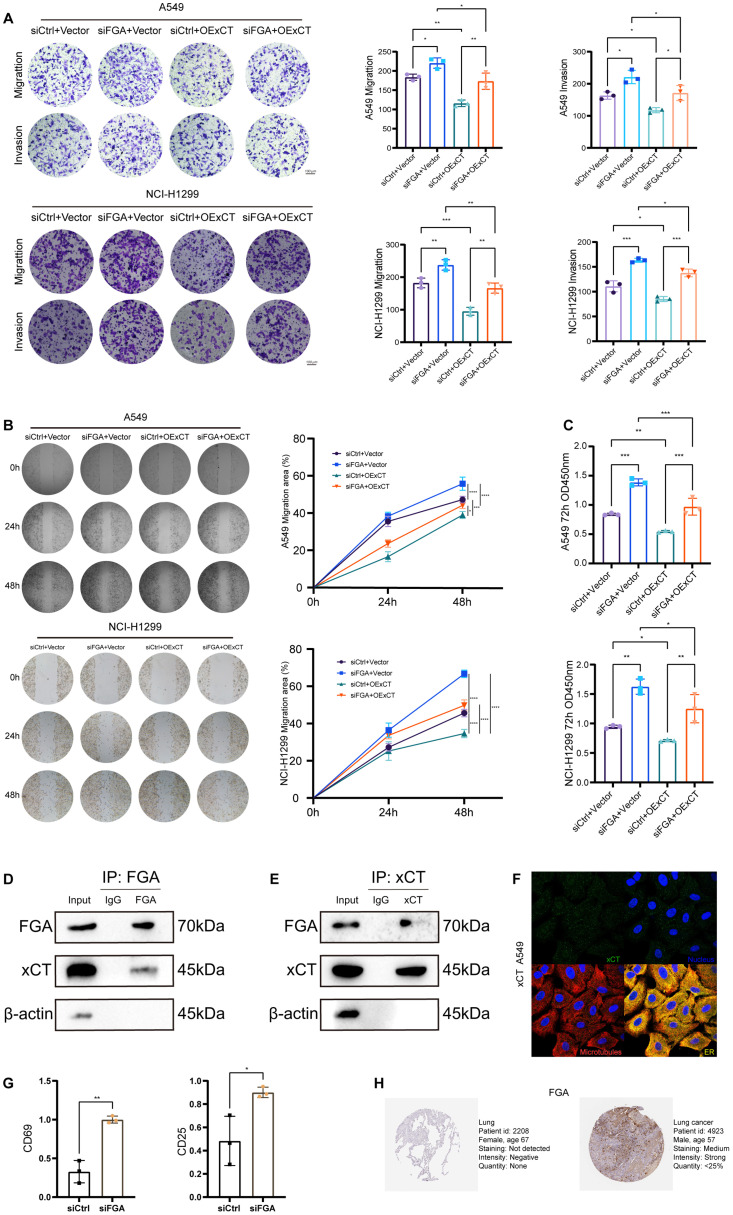
Protein-protein interaction between FGA and xCT modulates progression and immune evasion in lung cancer. **(A)** Transwell migration and invasion assays were performed in a rescue experiment. **(B)** Wound healing assays were performed to detect cell migration in the rescue experiment. **(C)** Cell proliferation in the rescue experiment after 72h was detected by CCK8. **(D)** Endogenous FGA co-immunoprecipitates xCT in A549 cells. **(E)** Endogenous xCT co-immunoprecipitates FGA in A549 cells. **(F)** Immunostaining for xCT in A549 cell from the Human Protein Atlas. **(G)** Tumor cell FGA deficiency potentiates CD8^+^ T-cell activation via soluble factors in a non-contact Transwell co-culture system. **(H)** Immunohistochemical staining of FGA proteins in normal tissues and lung cancer from the Human Protein Atlas. *P < 0.05, **P < 0.01, ***P < 0.001, ****P < 0.0001.

To mechanistically define the tumor suppressor function of FGA dependent on xCT, functional phenotypic assays were employed. Functional characterization in both A549 and NCI-H1299 cells demonstrated that xCT overexpression attenuated the phenotypes induced by FGA knockdown; xCT overexpression partially diminished the enhanced effects of FGA knockdown on cell migration and invasion (**Figure 7A**). Similarly, xCT overexpression partially counteracted the enhanced effects of FGA knockdown on migration of A549 and NCI-H1299 cells, as demonstrated in a wound-healing assay (**Figure 7B**). Additionally, CCK8 assay results showed that in A549 and NCI-H1299 (**Figure 7C**) cells, the stimulatory effect of FGA knockdown on cell proliferation was partially reversed by restoring xCT expression. Taken together, these results suggest that FGA through xCT inhibited tumor progression by suppressing cell viability, formation, migration, invasiveness, and tumor growth of lung cancer cells.

To explore the undefined molecular mechanism by which FGA regulates xCT in LUAD cells, we performed co-immunoprecipitation (co-IP) assays to determine physical interaction between these proteins. For all co-IP experiments, whole-cell lysates were used as positive controls in immunoblotting. Normal mouse IgG served as negative controls matched for isotype in immunoprecipitation. Reciprocal co-immunoprecipitation assays in A549 cells confirmed a specific physical interaction between FGA and xCT. Endogenous FGA immunoprecipitates pulled down xCT from whole-cell lysates (**Figure 7D**), while reverse IP with anti-xCT antibodies co-precipitated FGA (**Figure 7E**). Collectively, reciprocal co-immunoprecipitation analyses demonstrate that endogenous FGA and xCT form a protein complex in A549 cells. At the subcellular level, FGA resides in the endoplasmic reticulum as a secretory protein, while xCT resides in vesicles as a membrane transporter protein (**Figure 7F**). Source: The Human Protein Atlas. This physical interaction mechanistically underlies FGA-driven disulfidptosis regulation in LUAD.

To investigate the impact of tumor cell FGA on CD8^+^ T-cell activation, we employed a non-contact Transwell co-culture system. Following 48h of co-culture in a non-contact Transwell system, where pre-activated CD8^+^ T cells (upper chamber) were exposed to soluble factors from either control or siFGA A549 cells (lower chamber), qPCR analysis of the CD8^+^ T cells co-cultured with siFGA A549 cells significantly enhanced the expression of activation markers CD69 (*p* < 0.01) and CD25 (*p* < 0.05) (**Figure 7G**) in CD8^+^ T cells compared to the control group. This augmented expression of T-cell activation markers suggests an enhanced anti-tumor immune response in the context of tumor cell FGA deficiency.

The Human Protein Atlas contains extensive transcriptomics and proteomics data for specific human tissues and includes the Tissue Atlas, Cell Atlas, and Pathology Atlas. In this study, we examined the protein expression of the FGA gene in normal lung tissues and lung cancer tissues using this database, and the results suggest that FGA is decreased in normal tissue and increased in lung cancer tissues (**Figure 7H**). This spatial compartmentalization pattern, integrated with phenotypic rescue evidence, delineates a mechanistic framework wherein FGA within the TME suppresses oncogenesis and influences immune infiltration through xCT-mediated regulation of disulfidptosis.

## Discussion

In recent years, there has been an increased focus on the metabolism of tumor and immune cells in the hypoxic and nutrient-depleted TME. Malignant cell metabolic alterations, driven by intrinsic and extrinsic mechanisms, disrupt innate and adaptive immunity while accelerating disease progression (22, 23). These metabolic perturbations further induce energy expenditure-metabolite production imbalances in the tumor microenvironment (TME), subsequently disrupting signal transduction and gene expression to create an immunosuppressive environment that aids in tumor growth (24). Understanding the diversity and complexity of the tumor immune microenvironment and its regulatory mechanisms’ relationship with therapeutic approaches is significant. While cancer immunotherapy demonstrates therapeutic efficacy, persistent clinical challenges remain. Suboptimal delivery kinetics, for instance, contribute to diminished treatment responses across diverse tumor types (25). Therefore, novel immunotherapeutic strategies need to be developed, and understanding the mechanisms driving disulfidptosis may provide new therapeutic avenues. Through screening of genes associated with disulfidptosis and immune infiltration combined with bioinformatics analysis and experimental validation, we discovered that the fibrinogen alpha chain (FGA) modulates xCT expression via protein-protein interaction to regulate disulfidptosis, thereby influencing tumor progression and immune infiltration within the TME during LUAD development.

The fibrinogen is comprised of FGA, beta chain (FGB), and gamma chain (FGG). Research indicates that fibrinogen significantly contributes to tumor cell growth, the epithelial-to-mesenchymal transition, invasion, angiogenesis, and the spread of tumor cells through the bloodstream (26). Emerging evidence has demonstrated that FGA functions as a tumor suppressor, whose genetic mutations could promote hepatocarcinogenesis through disruption of its tumor-suppressive regulatory mechanisms (16). In this study, our findings provide evidence that FGA plays a role in immune cell infiltration and the formation of immunotherapeutic responses in LUAD, while also identifying its tumor-suppressive role. Functional studies demonstrated that knockdown of FGA promotes lung cancer cell proliferation, migration, and invasion in a low glucose environment by reducing xCT expression levels, highlighting its important role in disulfidptosis. Within the tumor microenvironment (TME), where myeloid and T cells demonstrate preferential glucose uptake over malignant cells (14), the secreted protein FGA orchestrates tumor matrix remodeling and immune response modulation through xCT expression regulation, thereby shaping clinical prognosis. Additional studies are also needed to understand how the FGA affects the establishment and maintenance of a tumorigenic niche, as well as its effect on tumor stromal cells and the immune microenvironment.

SLC7A11 (also known as xCT), significantly upregulated in various human malignancies and crucial for tumor initiation, development, and drug resistance (27, 28), exhibits a dual regulatory role by governing both ferroptosis and disulfidptosis. This gene can induce distinct cell death modes under varying conditions, reflecting the interconnected network among cell death pathways (29–33). While accumulating evidence shows that SLC7A11 is precisely regulated at both transcriptional and post-translational levels, the specific transcription, post-transcription, and post-transcriptional modifications of SLC7A11 in lung cancer remain unclear. In this study, we have identified the FGA protein as a significant regulator of the SLC7A11 transporter. Disulfidptosis is a novel mechanism of cell death that has potential applications in cancer treatment and other diseases (13, 34). Our multi-omics study suggests that FGA modulates xCT expression and inhibits GLUT to induce disulfidptosis, supporting its feasibility as a therapeutic target (20, 35–38). Given the complex interplay of signaling pathways, tumor microenvironment (TME) alterations, and regulated cell death modes in tumorigenesis, future studies should adopt integrated approaches that combine transcriptomics, proteomics, and computational models. These approaches should aim to mitigate biases and validate findings in additional lung adenocarcinoma (LUAD) cell lines, as well as relevant immune cell co-culture models, to enhance model generalizability (39, 40). Our *in-vivo* xenograft studies demonstrated that FGA knockdown significantly accelerated tumor growth in immunodeficient mice, suggesting FGA suppresses LUAD progression. Furthermore, future studies utilizing patient-derived xenograft (PDX) models preserving native tumor heterogeneity are warranted to validate these findings in a more clinically relevant context (41–43). Crucially, current techniques for inducing disulfidptosis remain limited. Overcoming this barrier is vital for therapeutic translation; future strategies should focus on developing agents that either deplete intracellular NADPH or directly induce disulfide bond stress to trigger effective tumor disulfidptosis. These critical advancements will pave the way for tailored treatments, meaning therapy specifically designed for each patient, rather than a blanket approach for all. This shift towards precision medicine promises more effective targeting of tumors while minimizing complications (44–46).

Collectively, our findings demonstrate that FGA modulates xCT expression via protein-protein interaction to play a critical role in mediating disulfidptosis within the TME of LUAD patients. These discoveries provide novel insights into the functional significance of disulfidptosis in shaping LUAD TMEs.

## Data Availability

The original contributions presented in the study are included in the article/**Supplementary Material**. Further inquiries can be directed to the corresponding author.
