# Correction: FGA modulates immune infiltration and tumor progression via SLC7A11/xCT-mediated disulfidptosis in the tumor microenvironment of lung adenocarcinoma

**DOI:** 10.3389/fimmu.2026.1789896

**Published:** 2026-02-17

**Authors:** Gen Li, Qiuping Li, Sheng Yang, Dongmei Guo, Yanling Tao, Yan Jia

**Affiliations:** 1 Department of Wound Reconstructive Surgery, Tongji Hospital, School of Medicine, Tongji University, Shanghai, China; 2 Department of Clinical Medicine, Jining Medical University, Jining, China; 3 Department of Hematology, Affiliated Hospital of Jining Medical University, Jining, China; 4 Weishan County People’s Hospital, Jining, China

**Keywords:** immune infiltration, disulfidptosis, tumor microenvironment, non-small-cell lung cancer, lung adenocarcinoma

There was a mistake in **Figure 7** as published. **Figure 7** was inadvertently overwritten by an incorrect image. The corrected **Figure 7** appears below.

**Figure 7:**
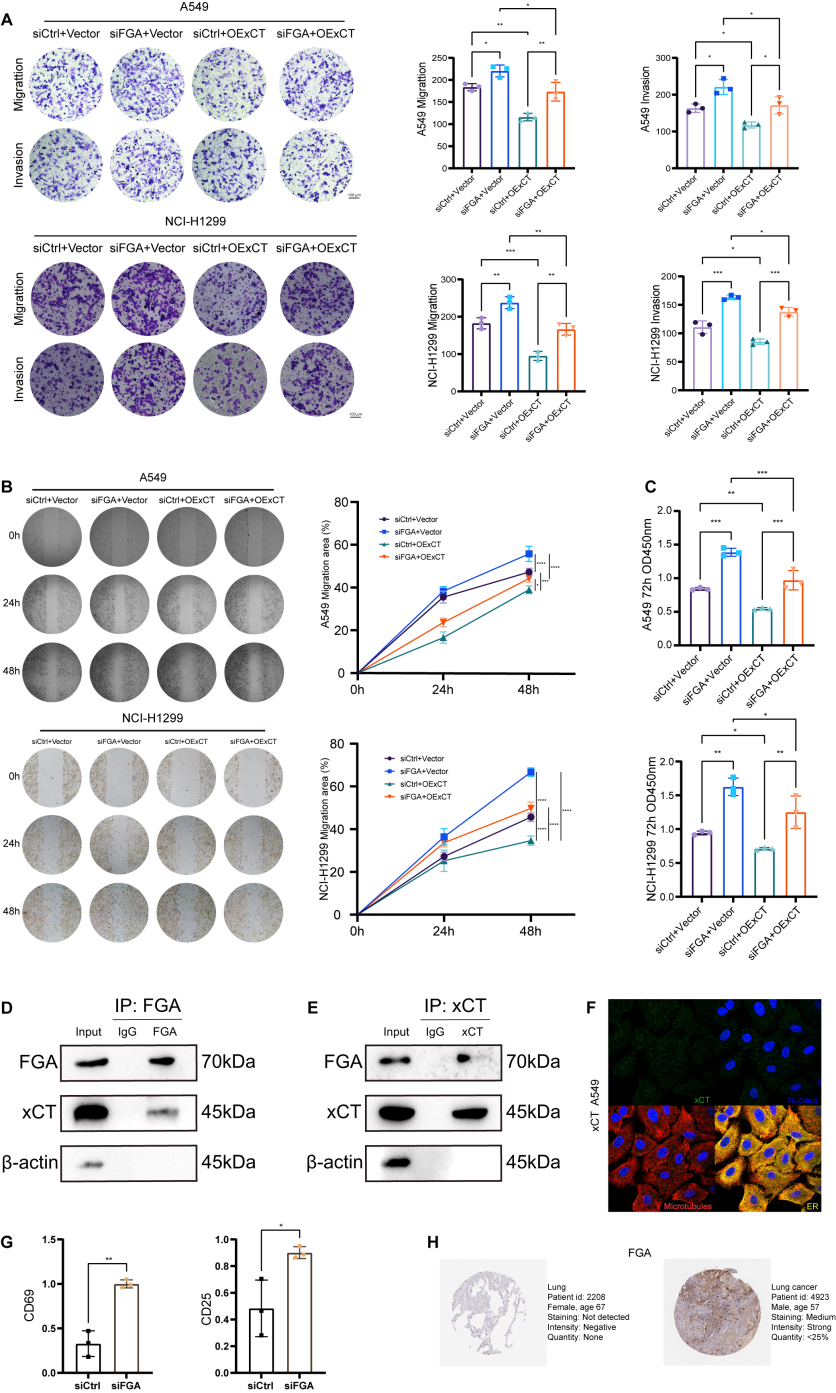
Protein-protein interaction between FGA and xCT modulates progression and immune evasion in lung cancer. **(A)** Transwell migration and invasion assays were performed in a rescue experiment. **(B)** Wound healing assays were performed to detect cell migration in the rescue experiment. **(C)** Cell proliferation in the rescue experiment after 72h was detected by CCK8. **(D)** Endogenous FGA co-immunoprecipitates xCT in A549 cells. **(E)** Endogenous xCT co-immunoprecipitates FGA in A549 cells. **(F)** Immunostaining for xCT in A549 cell from the Human Protein Atlas. **(G)** Tumor cell FGA deficiency potentiates CD8+ T-cell activation via soluble factors in a non-contact Transwell co-culture system. **(H)** Immunohistochemical staining of FGA proteins in normal tissues and lung cancer from the Human Protein Atlas. *P < 0.05, **P < 0.01, ***P < 0.001, ****P < 0.0001.

The original version of this article has been updated.

