# Supplementary figures and images for: FGA modulates immune infiltration and tumor progression via SLC7A11/xCT-mediated disulfidptosis in the tumor microenvironment of lung adenocarcinoma
Source: Front Immunol. 2025 Aug 11;16:1595900. doi: 10.3389/fimmu.2025.1595900 (PMC12375581; doi:10.3389/fimmu.2025.1595900)

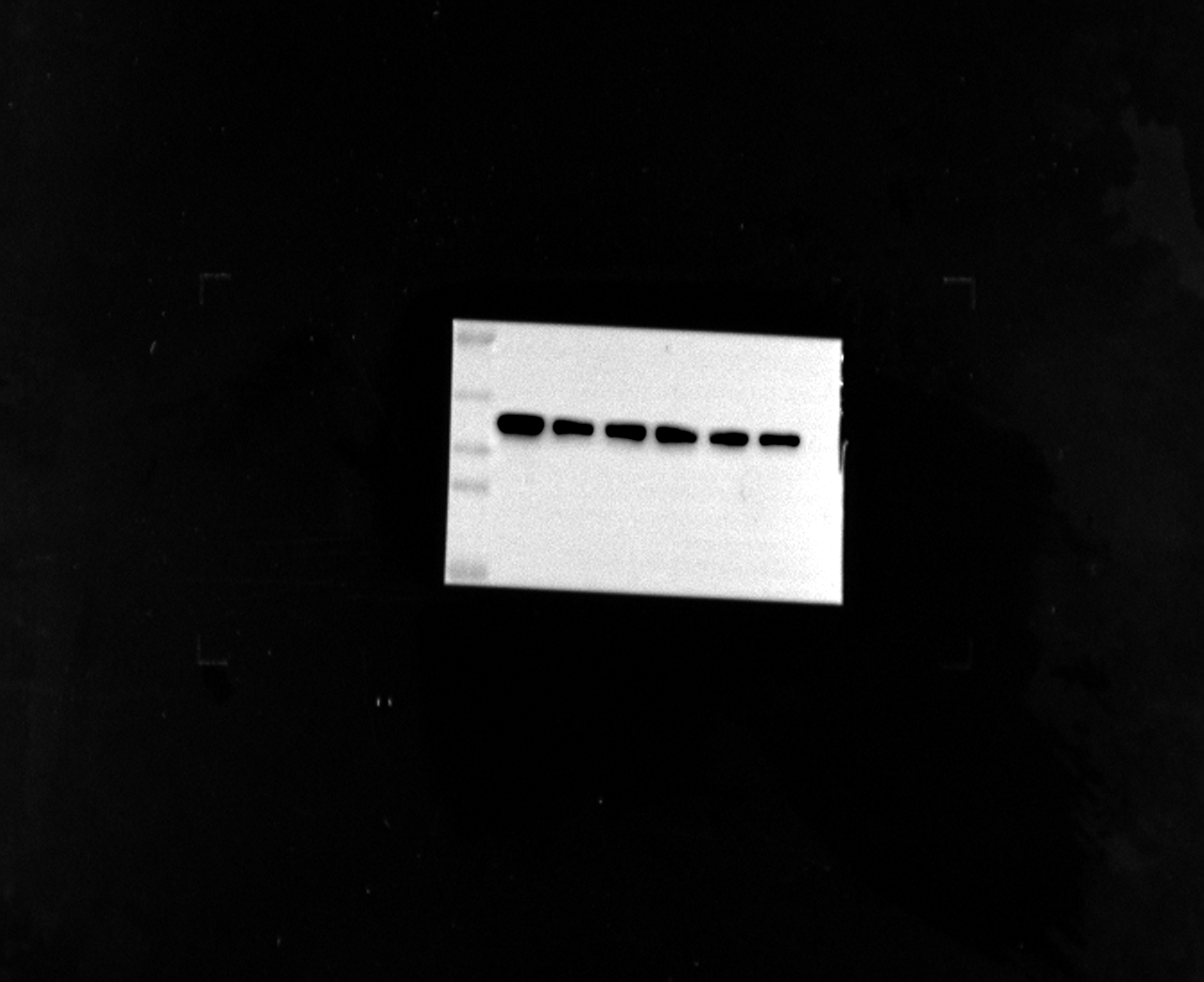

Supplement: Supplementary file 1 [file DataSheet1.zip › F5/WB/00actin merge 1.tif]

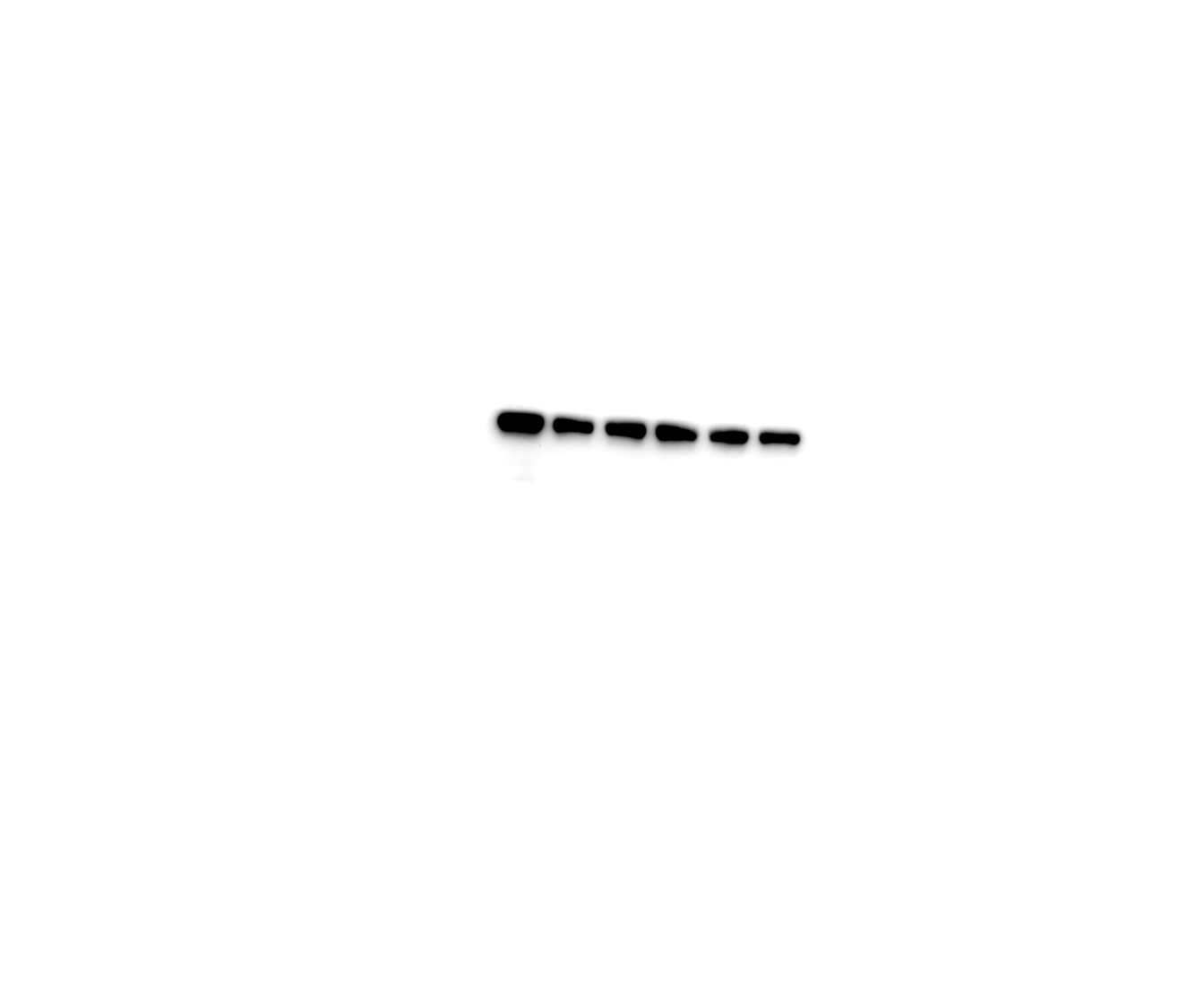

Supplement: Supplementary file 1 [file DataSheet1.zip › F5/WB/00actin-10S.tif]

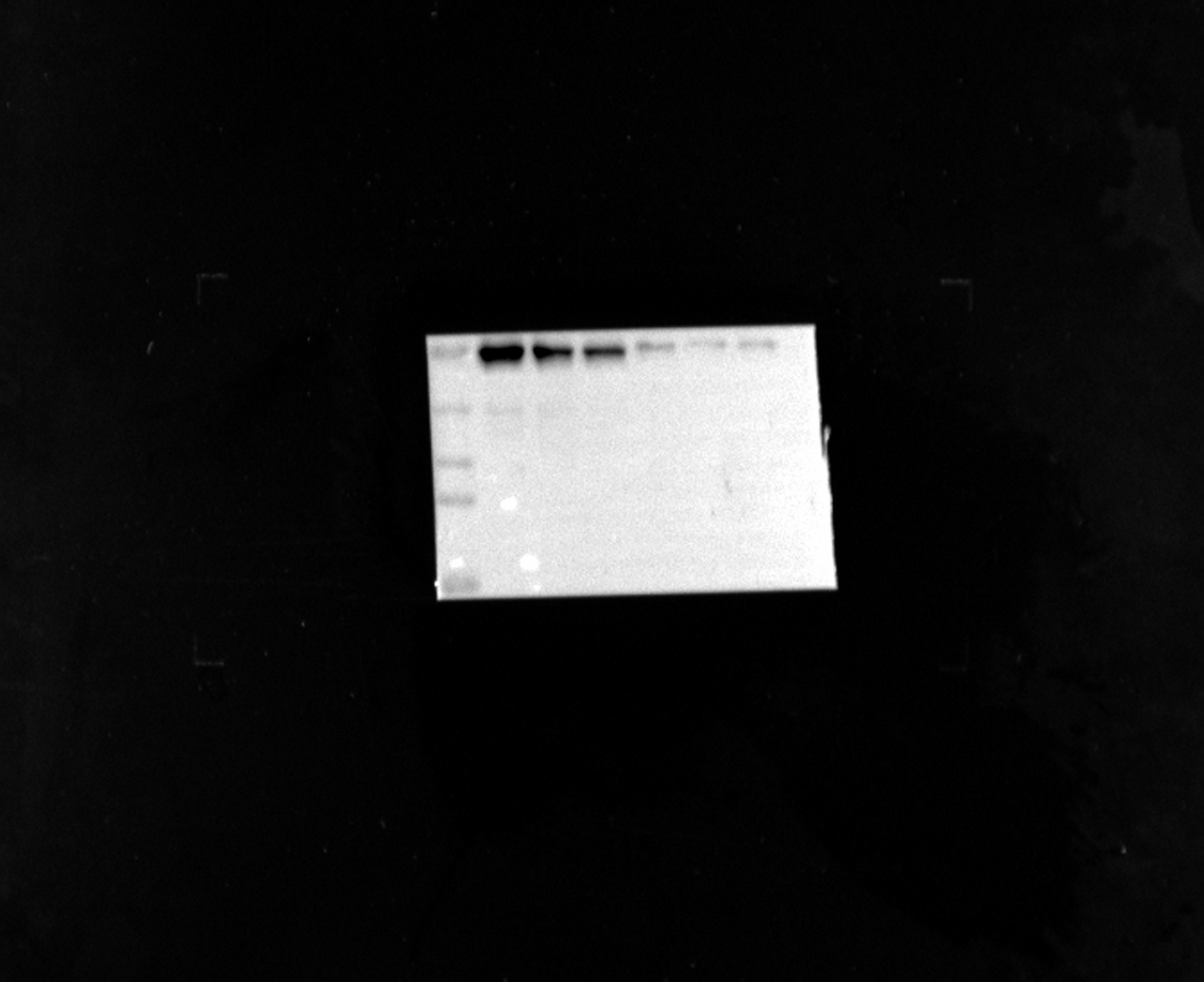

Supplement: Supplementary file 1 [file DataSheet1.zip › F5/WB/01膜4 FGA merge3.tif]

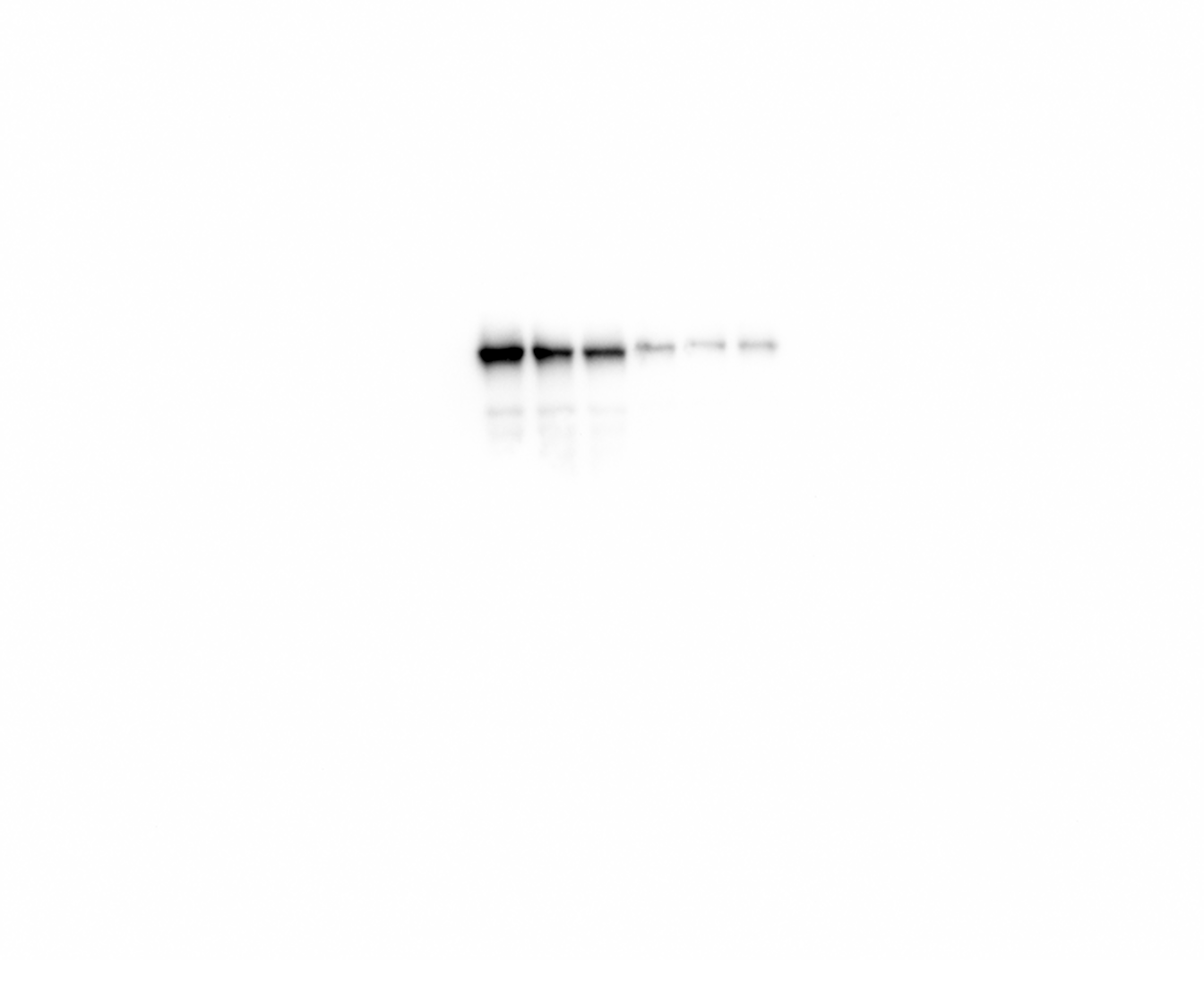

Supplement: Supplementary file 1 [file DataSheet1.zip › F5/WB/01膜4 FGA.tif]

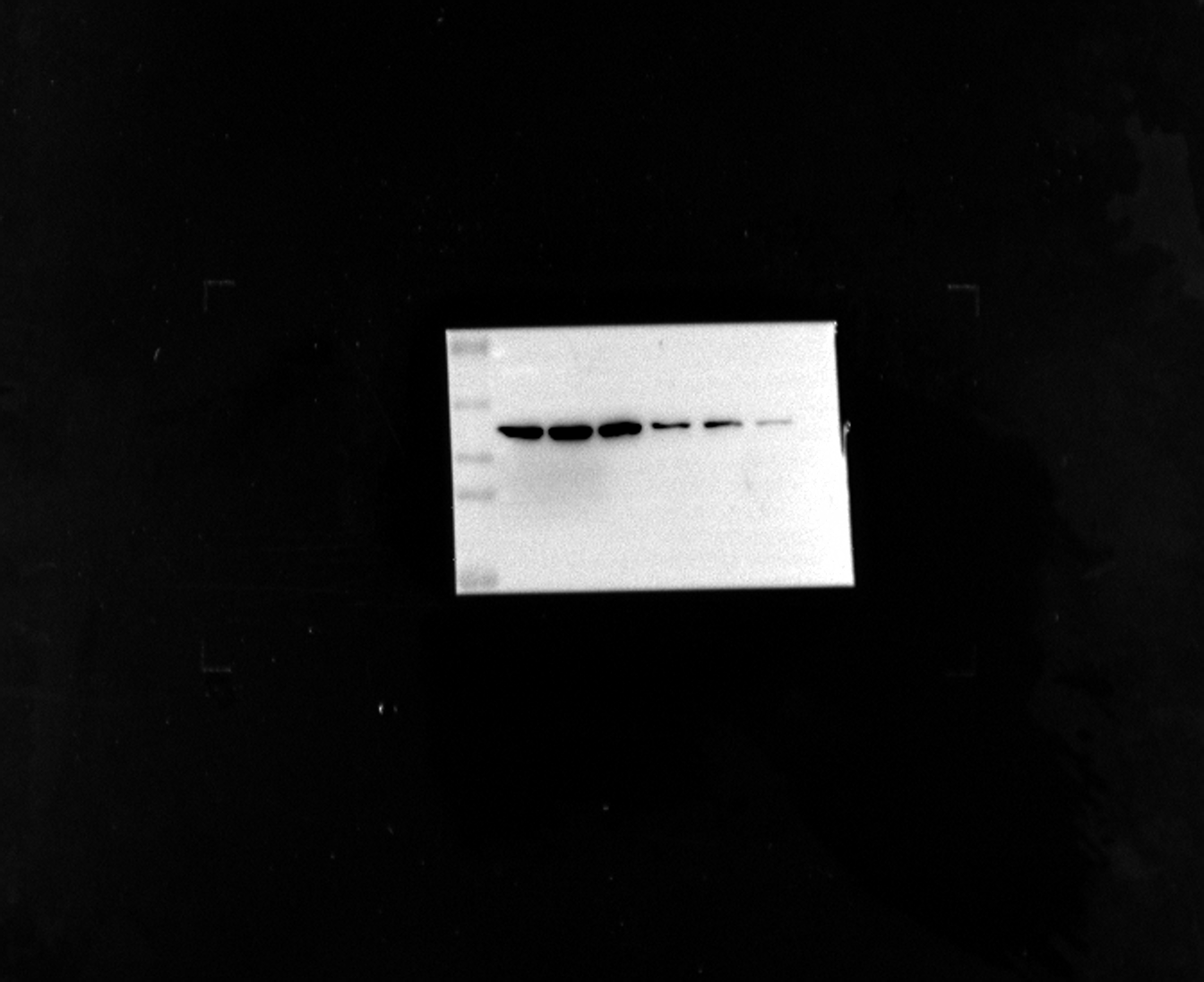

Supplement: Supplementary file 1 [file DataSheet1.zip › F5/WB/03xCTmerge3.tif]

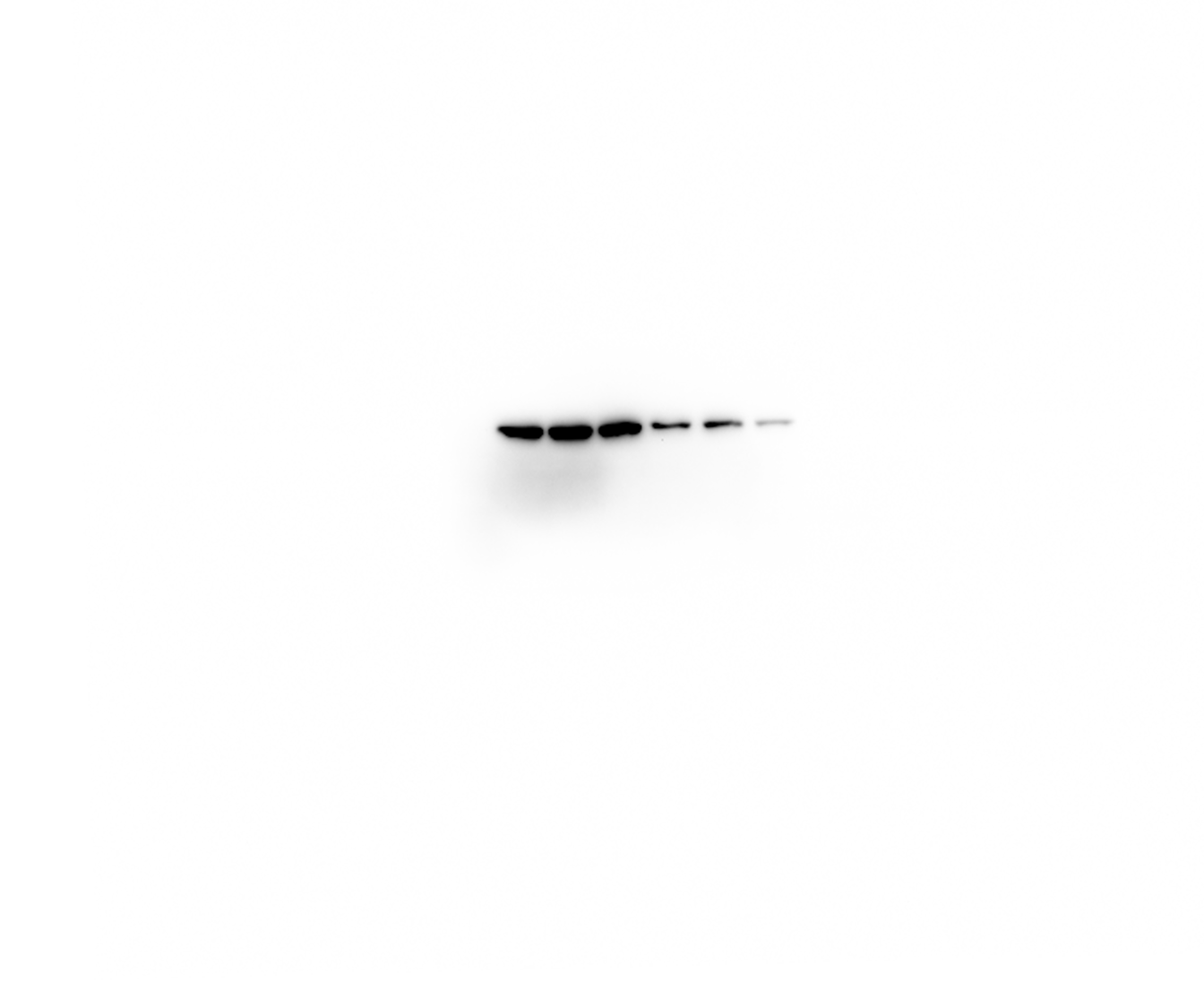

Supplement: Supplementary file 1 [file DataSheet1.zip › F5/WB/03xCTz-1.Tif]

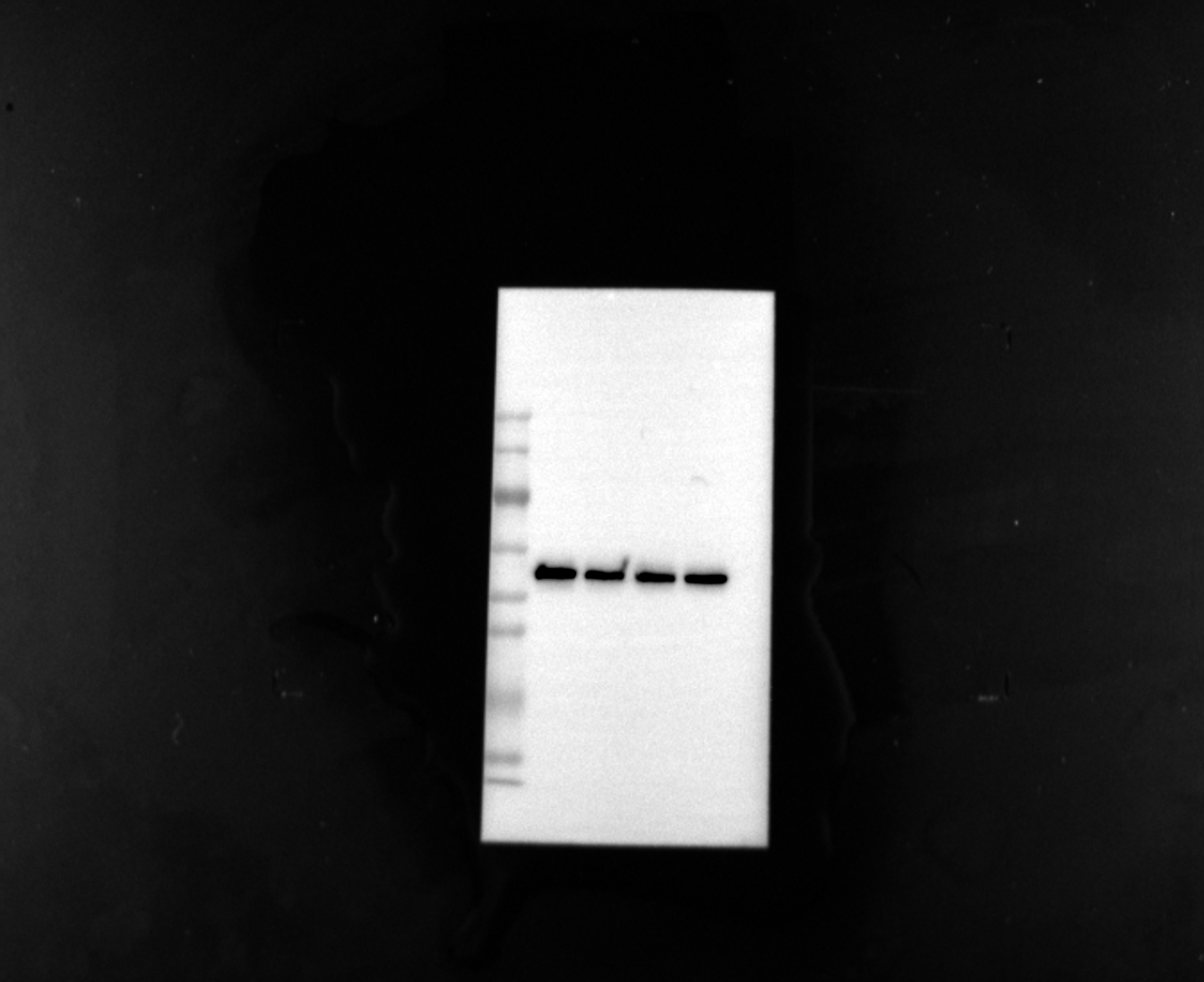

Supplement: Supplementary file 1 [file DataSheet1.zip › F6/WB/ACTIN/actin 3s merge.Tif]

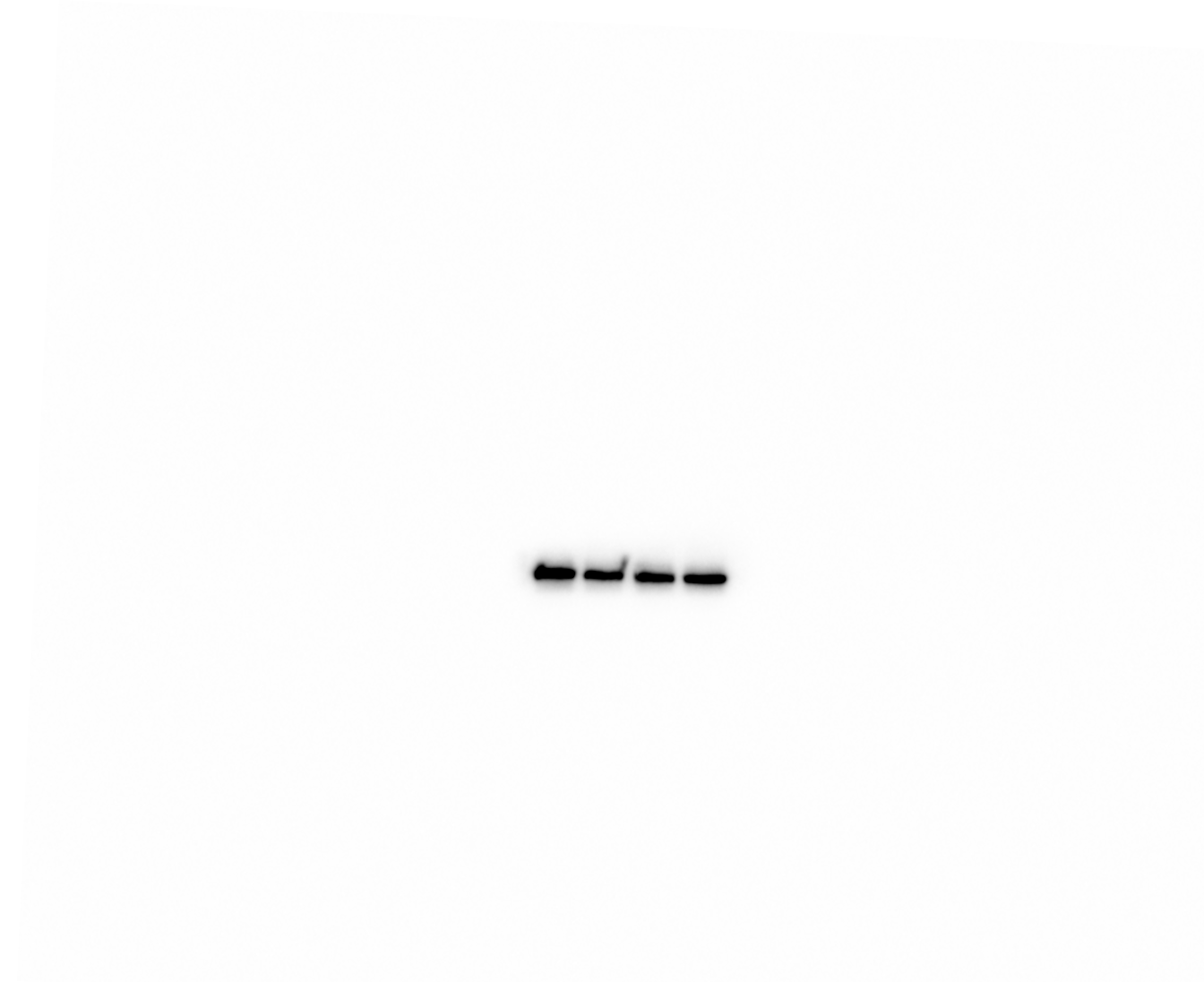

Supplement: Supplementary file 1 [file DataSheet1.zip › F6/WB/ACTIN/actin 3s.Tif]

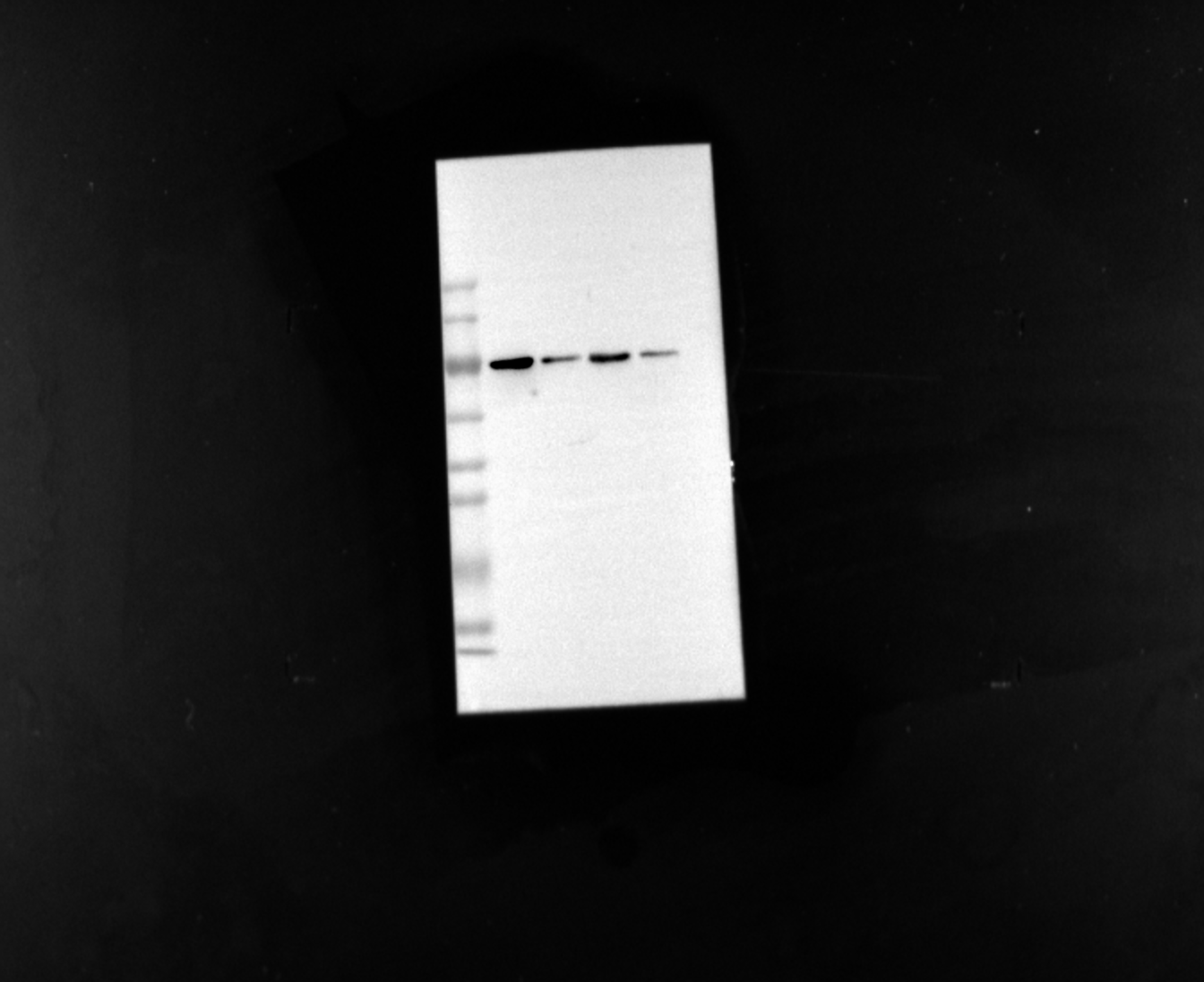

Supplement: Supplementary file 1 [file DataSheet1.zip › F6/WB/FGA/FGA10s 03 merge.Tif]

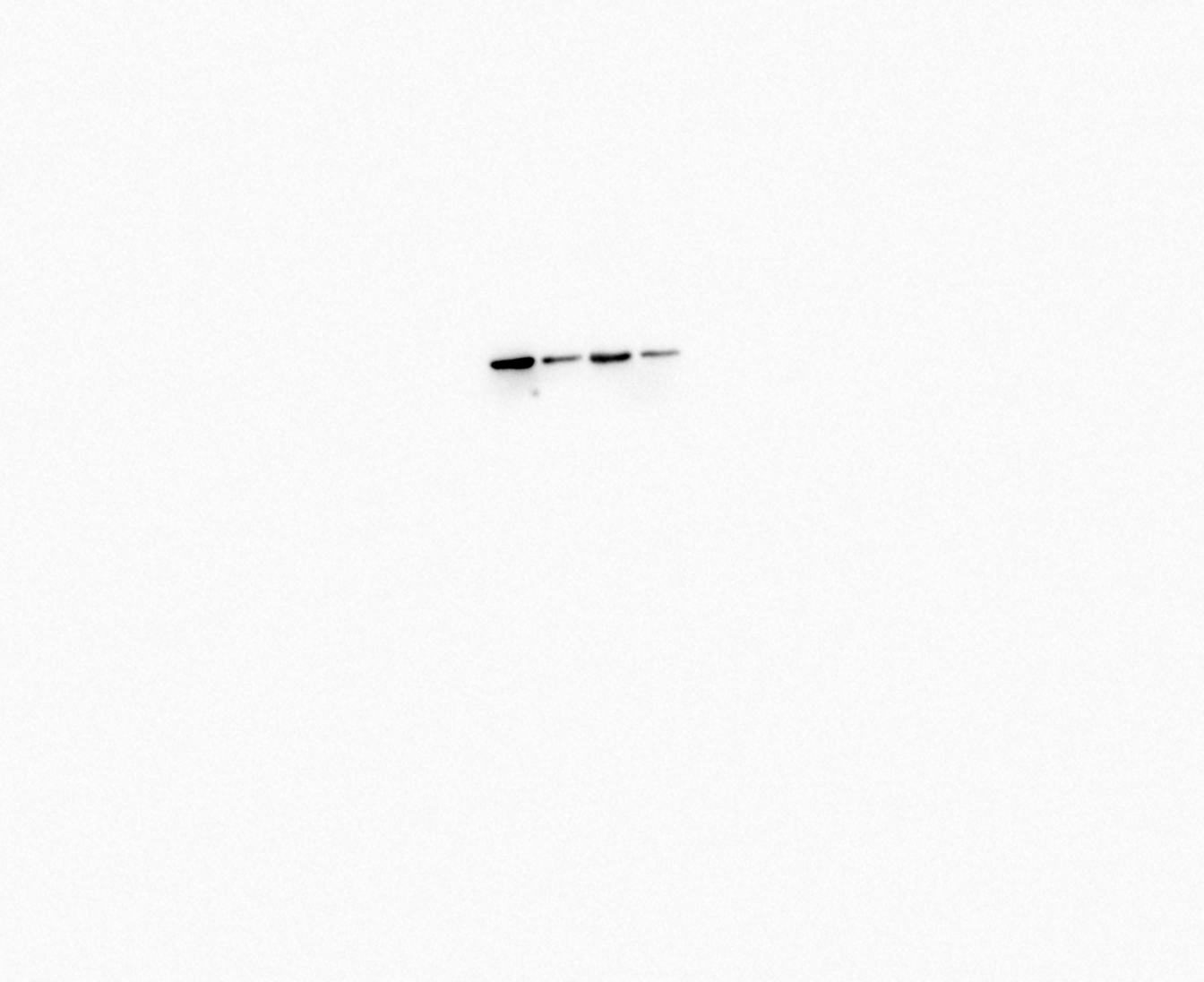

Supplement: Supplementary file 1 [file DataSheet1.zip › F6/WB/FGA/FGA10s 03.Tif]

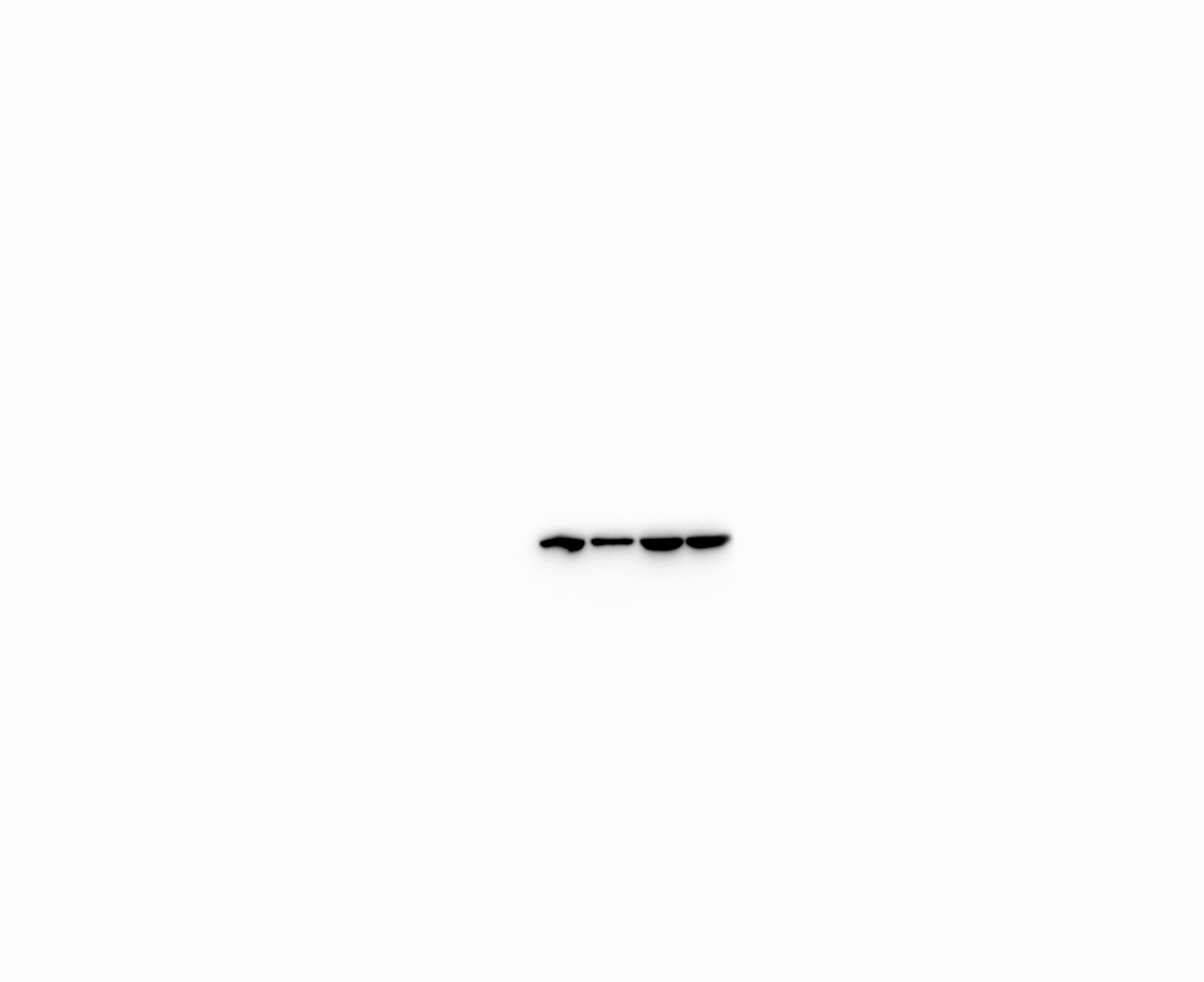

Supplement: Supplementary file 1 [file DataSheet1.zip › F6/WB/xCT/Xct 3s.Tif]

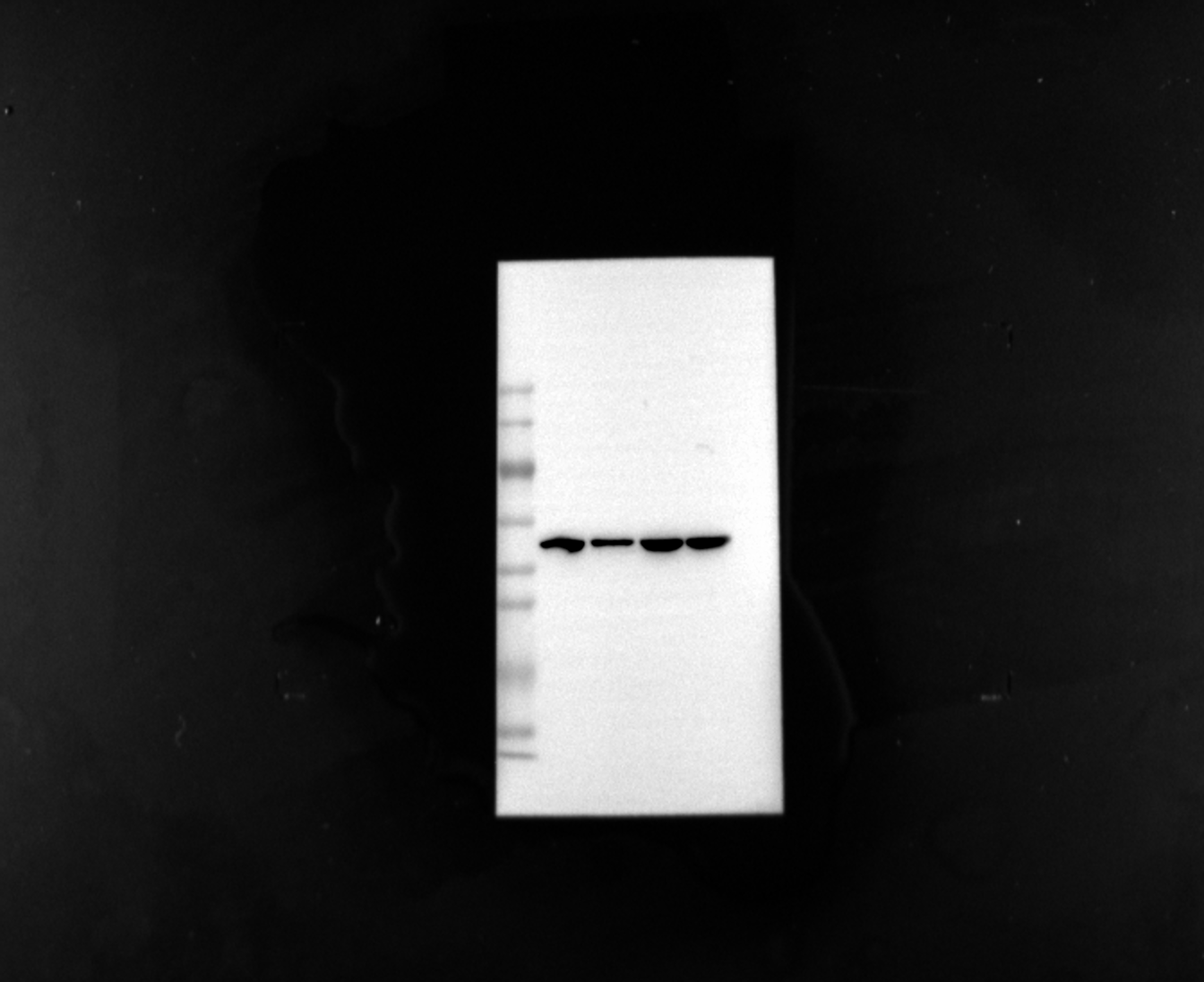

Supplement: Supplementary file 1 [file DataSheet1.zip › F6/WB/xCT/xCT merge 02.tif]

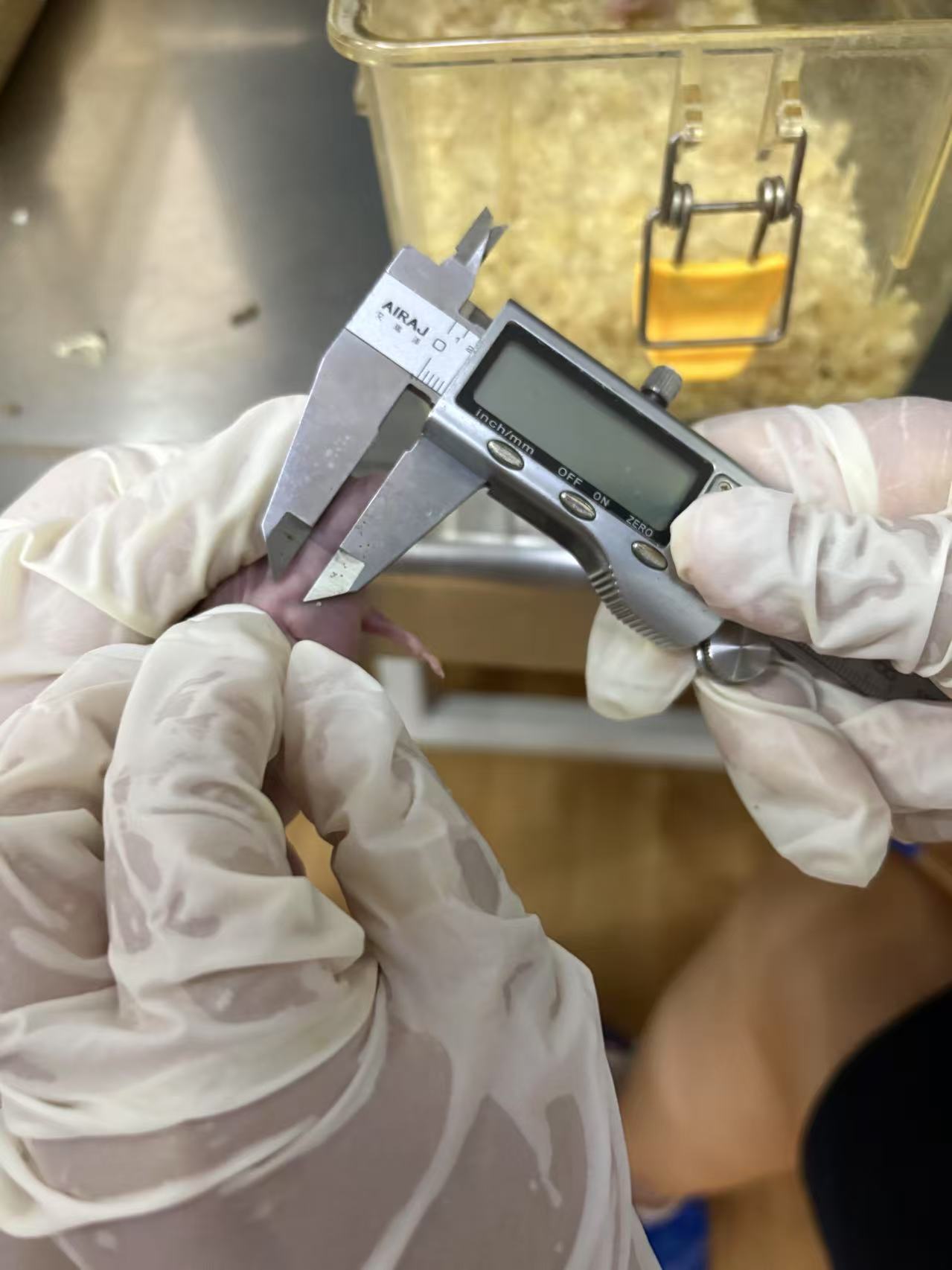

Supplement: Supplementary file 2 [file Image1.jpeg]

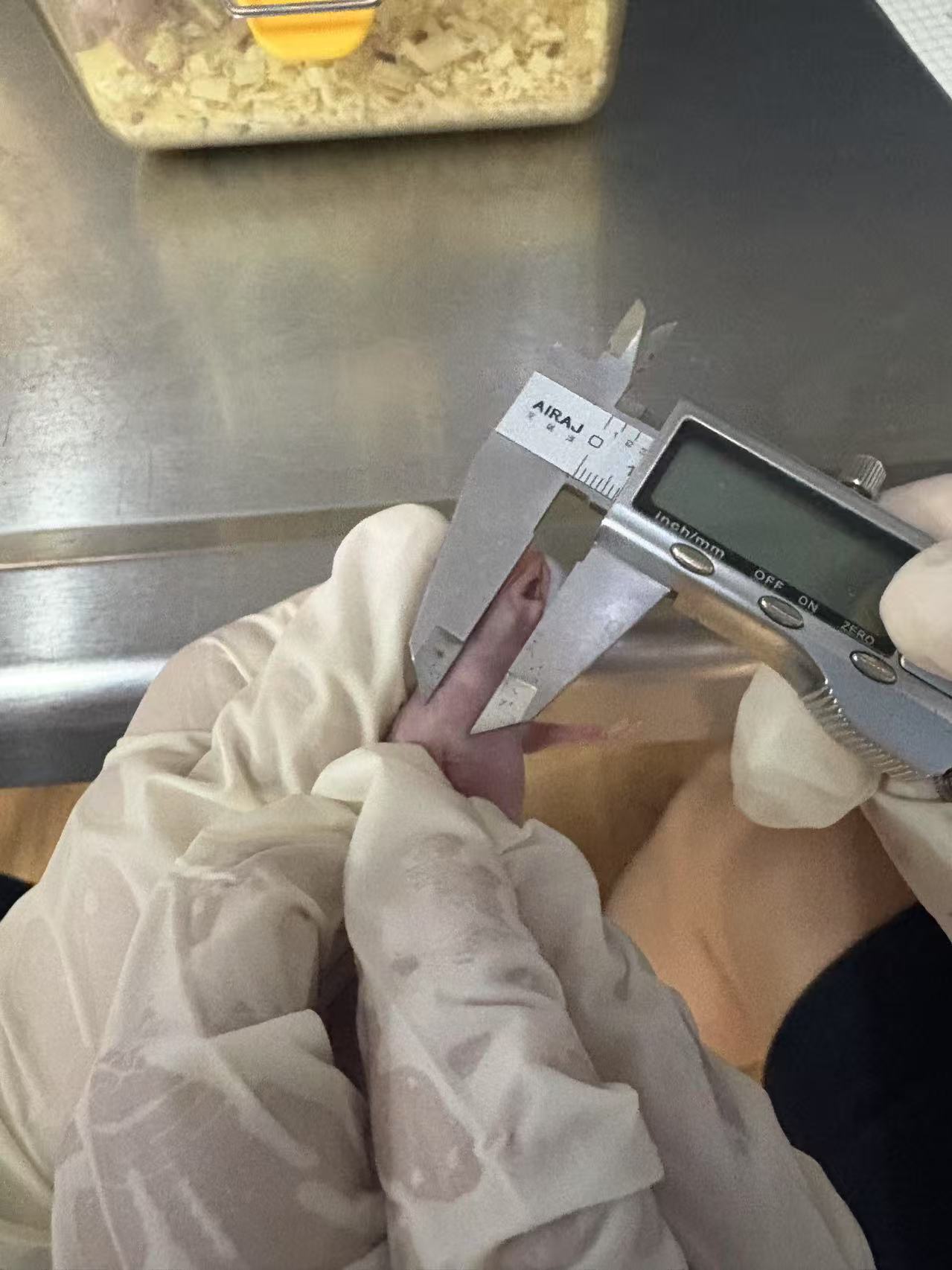

Supplement: Supplementary file 3 [file Image2.jpeg]
